# Functional connectivity correlates of reduced goal-directed behaviors in behavioural variant frontotemporal dementia

**DOI:** 10.1007/s00429-022-02519-5

**Published:** 2022-06-25

**Authors:** Valérie Godefroy, Bénédicte Batrancourt, Sylvain Charron, Arabella Bouzigues, David Bendetowicz, Guilhem Carle, Armelle Rametti-Lacroux, Stéphanie Bombois, Emmanuel Cognat, Raffaella Migliaccio, Richard Levy

**Affiliations:** 1 Inserm U 1127, CNRS UMR 7225, Sorbonne Université, Institut du Cerveau-Paris Brain Institute-ICM, Inserm, CNRS, AP-HP, Hôpital de la Pitié Salpêtrière, 47 Boulevard de l’Hôpital, 75013 Paris, France; 2 INSERM U1266, Institut de Psychiatrie et Neurosciences de Paris, Université de Paris, Paris, France; 3 Department of Neuroradiology, Hôpital Sainte-Anne, Université de Paris, Paris, France; 4 Department of Neurology, IM2A, AP-HP, Groupe Hospitalier Pitié-Salpêtrière, Paris, France; 5 Behavioural Neuropsychiatry Unit, AP-HP, Hôpital de la Salpêtrière, Paris, France; 6 UMRS 1144, INSERM, 5010, Université de Paris, Paris, France; 7 Centre de Neurologie Cognitive, Hôpital Lariboisière Fernand-Widal, APHP Nord, 75010 Paris, France

**Keywords:** Frontotemporal dementia, Apathy, Goal-directed behavior, Ecological design, fMRI, Resting-state functional connectivity

## Abstract

We explored the resting state functional connectivity correlates of apathy assessed as a multidimensional construct, using behavioral metrics, in behavioral variant frontotemporal dementia (bvFTD). We recorded the behavior of 20 bvFTD patients and 16 healthy controls in a close-to-real-life situation including a free phase (FP—in which actions were self-initiated) and a guided phase (GP—in which initiation of actions was facilitated by external guidance). We investigated the activity time and walking episode features as quantifiers of apathy. We used the means ((FP + GP)/2) and the differences (FP-GP) calculated for these metrics as well as measures by questionnaires to extract apathy dimensions by factor analysis. We assessed two types of fMRI-based resting state connectivity measures (local activity and seed-based connectivity) and explored their relationship with extracted apathy dimensions. Apathy in bvFTD was associated with lower time spent in activity combined with walking episodes of higher frequency, lower acceleration and higher duration. Using these behavioral metrics and apathy measures by questionnaires, we disentangled two dimensions: the global reduction of goal-directed behaviors and the specific deficit of self-initiation. Global apathy was associated with lower resting state activity within prefrontal cortex and lower connectivity of salience network hubs while the decrease in self-initiation was related to increased connectivity of parietal default-mode network hubs. Through a novel dimensional approach, we dissociated the functional connectivity correlates of global apathy and self-initiation deficit. We discussed in particular the role of the modified connectivity of lateral parietal cortex in the volitional process.

**Supplementary Information:**

The online version contains supplementary material available at 10.1007/s00429-022-02519-5.

## Background

Apathy is the most frequent behavioral syndrome in neurological and psychiatric diseases (Levy and Dubois 2006; Levy 2012; Le Heron et al. 2018) and is also prevalent in varying degrees in healthy people (Ang et al. 2017). In neurological diseases, apathy is considered as a major source of morbidity predicting bad prognosis (Levy 2012; Lansdall et al. 2019; Malpetti et al. 2021). Apathy is also associated with a higher level of global functional impairment and loss of autonomy (Lechowski et al. 2009; Wadsworth et al. 2012), and it has a negative impact on the quality of life of both patients and their caregivers (Hurt et al. 2008). Considering the globally high prevalence of apathy and the extent of its debilitating consequences, it is of high importance to advance our understanding of this syndrome with the final aim of improving its treatment.

Apathy is traditionally defined as a “lack of motivation not attributable to diminished level of consciousness, cognitive impairment or emotional distress’’ (Marin 1991, 1996). The clinical scales typically used to assess apathy (e.g., the Starkstein Apathy Scale—SAS (Starkstein et al. 1992)) are based on this definition and mostly rely on questions about a patient’s internal state of mind. They are, therefore, biased by the subjective perspective of the reporter (Levy and Dubois 2006; Levy 2012; Godefroy et al. 2020). In line with the recent international consensus on apathy criteria in neurodegenerative diseases (Robert et al. 2018), we define apathy as an observable quantitative reduction of goal-directed behaviors. For the present study, relying on this behavioral definition of apathy, we have developed new tools allowing us to objectively quantify goal-directed behaviors in semi-ecological conditions. This was a starting point towards a better understanding of the mechanisms involved in the quantitative production of goal-directed behaviors.

Several non-exclusive mechanisms may underlie the reduction of goal-directed behaviors observed in apathetic states (Massimo et al. 2014). Levy et al., (Levy and Dubois 2006; Levy 2012; Godefroy et al. 2020) have postulated the existence of three subtypes of apathy mechanisms (i.e., ‘emotional-affective’, ‘cognitive’, and ‘auto-activation’) corresponding to the disruption of different processes underpinned by distinct neural substrates. The ‘emotional-affective’ subtype corresponds to the disruption of emotional processing which may disrupt the motivation for goal-directed behaviors due to emotional desensitization to both positive and negative stimuli. In the ‘cognitive’ apathy subtype, goal-directed behaviors are reduced due to impaired cognitive functions needed to elaborate and implement a plan of actions. The ‘auto-activation’ subtype refers to difficulties in activating thoughts or initiating the motor program necessary to complete the behavior. In this model of apathy mechanisms, the emotional-affective subtype is assumed to be related to the “orbital-ventral-mesial circuit” linking the orbital and ventromedial PFC to the ventral regions of the basal ganglia. The cognitive subtype is attributed to damage to the “dorsolateral circuit” linking the DLPFC to the dorsal regions of the basal ganglia. Finally, the auto-activation deficit is associated with the “anterior cingulate-dorsal mesial circuit” linking the ACC and DMPFC to basal ganglia portions located between the dorsal and ventral regions.

All the tools developed to assess apathy as a multidimensional construct, like the Philadelphia Apathy Computerized Test (Massimo et al. 2015) or the Dimensional Apathy Scale (DAS) (Radakovic and Abrahams 2014), dissociate these three aspects of apathy although they are potentially concurrent and overlapping (Ducharme et al. 2018). Besides, several authors (Levy and Dubois 2006; Levy 2012; Ducharme et al. 2018; Le Heron et al. 2018; Godefroy et al. 2020) agree on the assumption that a deficit of self-initiation of goal-directed behaviors, potentially reversed by external guidance (i.e., the external attribution of a goal), is a central feature to explain the global reduction of goal-directed behaviors observed in the apathy syndrome. This type of deficit can be illustrated by the case of patients with bilateral basal ganglia lesions resulting in a syndrome of auto-activation deficit (Laplane and Dubois 2001). In these patients, despite the loss of self-initiated behaviors, the ability to execute externally driven behavior is relatively spared (Schmidt et al. 2008).

Neurodegenerative dementias, in particular within the frontotemporal dementia (FTD) spectrum (including the behavioral variant [bvFTD]), are very interesting lesion models to investigate the neural correlates of apathy, and they can serve to understand apathy in other neuropsychiatric disorders (Ducharme et al. 2018). FTD is related to predominant degeneration of the prefrontal and temporal regions. BvFTD, the most common clinical variant of FTD, is characterized by significant changes in personality and behavior. Contrary to other language variants, it is associated with greater atrophy in frontal and insular regions than in temporal regions (Karageorgiou and Miller 2014; Chare et al. 2014). The main clinical symptoms observed in bvFTD patients are behavioral disinhibition, apathy and loss of empathy (Rascovsky et al. 2007, 2011). The presence of apathy is one of the most frequent criteria enabling a clinical diagnosis of bvFTD (Rascovsky et al. 2007, 2011) and it remains almost constant throughout the disease (Pasquier et al. 1999). According to a literature review (Ducharme et al. 2018), apathy in FTD is most robustly associated with atrophy, hypometabolism and hypoperfusion in the dorsolateral prefrontal cortex (DLPFC), the anterior and middle cingulate cortex (ACC and MCC), the orbitofrontal cortex (OFC) and the medial superior frontal gyrus (SFG). However, most studies that have investigated the neural correlates of apathy in FTD assessed apathy as a unidimensional construct using clinical scales. Some (Massimo et al. 2015; Kumfor et al. 2018; Wei et al. 2020), but this literature remains sparse, have explored the neural bases of apathy as a multidimensional concept.

Aside from atrophy, hypometabolism and hypoperfusion in specific brain regions, the modified resting state functional connectivity bears great potential in patients with dementia to explain their neuropsychiatric symptoms (Zhou and Seeley 2014). Functional connectivity is defined as the temporal dependency of neuronal activation patterns of anatomically separated brain regions driven by low-frequency fluctuations (from 0.01 to 0.1 Hz) in the blood oxygen-level-dependent signal (van den Heuvel and Hulshoff Pol 2010). The study of resting state functional connectivity has allowed to evidence that the brain contains discernable functional communities called resting-state networks which show within-community, high-level functional coupling. To our knowledge, only three studies (Day et al. 2013; Farb et al. 2013; Zhou et al. 2020) related functional connectivity to apathy (measured as a unidimensional concept by questionnaires) in FTD patients. Using different measures of functional connectivity, these three studies found links between apathy and the connectivity of the salience network (SN) and two of them (Farb et al. 2013; Zhou et al. 2020) observed a relationship with the connectivity of the default-mode network (DMN). These two resting state networks are both functionally related to goal-directed behaviors. The SN plays an important role in the processing of diverse emotionally significant internal and external stimuli and is therefore useful to adapt behavior according to a specific context (Seeley et al. 2012). The DMN supports a ‘‘default mode’’ of brain function (when an individual is awake and alert but not actively involved in a goal-directed task) and is deactivated upon initiation of a goal-directed behavior (Raichle et al. 2001). Evidence to date suggests that bvFTD mainly targets the SN, which would subsequently cause an enhancement of DMN connectivity, following the hypothesis that a lesion to either network should heighten activity and connectivity in the other network (Zhou et al. 2010). Thus, the clinical severity of bvFTD symptoms would be associated with reduced SN connectivity but enhanced DMN connectivity (Zhou et al. 2010).

In this study, to address the issue of using only subjective measures of apathy by clinical scales, we first aimed to identify objective behavioral markers associated with apathy in bvFTD using an ecological approach. We used an original paradigm capturing participants’ behavior (through sensor and video recording) in a close-to-real-life situation and we investigated the hypothesis that several behavioral metrics could be used as quantifiers of goal-directed behaviors contributing to assess apathy. In this particular ecological setting, we assumed that the total time spent in goal-directed behaviors and characteristics of walking, while moving in the room could be such quantifiers of goal-directed behaviors. Indeed, the selected characteristics of walking episodes were supposed to reflect the extent to which movements in the room were oriented towards specific goals (instead of being mere wanderings).

The second aim of this study was to tackle another issue related to the assessment of apathy. As the deficit of self-initiation of goal-directed behaviors is theorized to be one of the central mechanisms of apathy, disentangling the assessment of the global reduction of goal-directed behaviors (in other words, the severity of apathy syndrome) from the assessment of the specific self-initiation deficit (in other words, the subtype of apathy as a unique dimension) could be useful. Our ecological paradigm of behavior tracking included both a ‘free phase’ in which goal-directed behaviors were totally self-initiated and a ‘guided phase’ in which initiation was facilitated by external guidance. Thus, we could calculate for each quantifier of goal-directed behaviors: first, the mean on the two phases as a potential indicator of the global reduction of goal-directed behaviors; second, the difference between the two phases as a potential indicator of the specific deficit of self-initiation. Indeed, the more participants are characterized by a specific deficit of self-initiation underlying apathy, the more their markers of goal-directed behaviors should improve in guided phase compared to free phase.

The third objective of the study responded to the need to further investigate the functional connectivity correlates of apathy in bvFTD. For this purpose, we used the two dimensions of apathy (global apathy and specific self-initiation deficit), which we related to two measures of resting state connections: (1) the voxelwise fractional amplitude of low-frequency fluctuation (fALFF), used as a proxi measure for the local health of resting-state networks (Zou et al. 2008); (2) seed-based connectivity maps, indicators of the distant functional connectivity between chosen regions of interest (ROI) and the rest of the brain. These two measures of connectivity were complementary for two reasons. First, one was a measure of local connectivity, while the other measure assessed distant connectivity, which allowed to investigate how information processing for goal-directed behaviors arises from both interactions between adjacent brain areas and from distant projections across distributed brain systems (Sepulcre et al. 2010). Second, one measure (fALFF) was used for an exploratory strategy across the whole brain, whereas the other measure (seed-based) was used for a hypothesis-driven analysis. One of the originalities in our study of connectivity related to apathy in FTD (compared to the three previous studies Day et al. 2013; Farb et al. 2013; Zhou et al. 2020)) was that we investigated seed-based connectivity using seeds defined a priori: the hub regions of the SN and DMN, relying on previous results on apathy in FTD (Day et al. 2013; Farb et al. 2013; Zhou et al. 2020) and according to a model of connectivity changes in bvFTD patients elaborated by Zhou, Seeley and colleagues (Zhou et al. 2010; Zhou and Seeley 2014). The central nodes of the SN include the anterior insula (AI), considered as an afferent salience structure, and the anterior cingulate cortex (ACC), considered as an efferent visceromotor region (Zhou et al. 2010). Moreover, the DMN includes as main hubs: the precuneus/posterior cingulate cortex (PCC), medial prefrontal cortex (MPFC), and bilateral parietal cortices (LPC) (Raichle et al. 2001).

## Materials and methods

### Participants and protocol overview

Twenty bvFTD patients (35% women; mean age = 65.8 $\pm$ 8.8; age range = [45–82]) and 16 healthy controls (62.5% women; mean age = 62.9 $\pm$ 7.6; age range = [46–71]) matched to patients for age, gender and education level were included in this study. Further details on the demographic and neuropsychological characteristics of bvFTD patients and controls are displayed in Table 1. BvFTD patients were recruited in two tertiary referral centers, at the Pitié-Salpêtrière Hospital and the Lariboisière Fernand-Widal Hospital, in Paris. They were diagnosed according to the International Consensus Diagnostic Criteria (Rascovsky et al. 2011). To respect inclusion criteria, bvFTD patients had to present a Mini-Mental State Evaluation (MMSE) score of at least 20 (to make sure that they were able to undergo the full protocol). Healthy controls were recruited by public announcement. They had to present a MMSE score of at least 27.

**Table 1:** Demographical, cognitive and apathy characteristics of bvFTD patients and HC

	bvFTD	HC	bvFTD vs HC
% Women	35%	62.5%	${\chi }^{2}$= 1.71; *P* = 0.19
Age	65.8 (8.8)	62.9 (7.6)	*T* = 1.07; *P* = 0.29
Years since 1st symptoms	4.4 (2.3)	–	–
Education level	6.4 (2.0)	7.3 (1.1)	*W* = 111; *P* = 0.34
MMSE (/30)	24.1 (2.8)	29.4 (0.8)	*W* = 8; *P* < 0.001
DRS (/144)	119.5 (9.3)	142.3 (1.3)	*W* = 0; *P* < 0.001
FAB (/18)	12.5 (3.4)	17.3 (0.9)	*W* = 6; *P* < 0.001
SAS (/42)	15.4 (4.8)	6.3 (2.8)	*T* = 7.13; *P* < 0.001
DAS (/72)	30.4 (10.5)	19.6 (8.6)	*T* = 3.40; *P* < 0.01
DAS-Emotional (/24)	10.9 (3.9)	8.3 (3.4)	*T* = 2.15; *P* < 0.05
DAS-Initiation (/24)	9.9 (5.9)	7.0 (4.0)	*T* = 1.73*; P* = 0.09
DAS-Executive (/24)	9.7 (5.0)	4.3 (3.7)	*W* = 256*; P* < 0.01

Data are given as Mean (SD). BvFTD: *N* = 20/Controls: *N* = 16

MMSE, Mini-Mental State Examination; DRS, Dementia Rating Scale; FAB, Frontal Assessment Battery; SAS, Starkstein Apathy Scale; DAS, Dimensional Apathy Scale; n.s., non-significant

The data gathered for this article are part of the ECOCAPTURE protocol (Clinicaltrials.gov:NCT02496312; see Batrancourt et al. (2019) and Supplementary file 1 Part A for further details on this protocol) designed to investigate the behavioral signature and mechanisms of neuropsychiatric syndromes such as apathy or disinhibition (Godefroy et al. 2021). For this study, we used data allowing to investigate the behavioral signature and mechanisms of apathy: behavioral measures collected with an ecological approach, clinical measures of apathy (by questionnaires) and MRI data.

### Behavioral measures in a close-to-real life setting

#### The ECOCAPTURE setting

The ECOCAPTURE setting reproduced a close-to-real life situation (i.e., being left alone in a “waiting room”) with a predetermined script (see Supplementary file 1 Part B for further details on the ECOCAPTURE scenario), whereby participants were first left in a freely moving phase called “free phase” for 7 min. This phase was followed by other phases including a “guided phase” lasting 10 min. In guided phase, participants were asked by the experimenter to fill out a questionnaire, with very easy questions, but requiring in-depth exploration of the room. Thus, in the free phase, participants had to self-initiate goal-directed behaviors whereas in the guided phase, the initiation of goal-directed behaviors was facilitated by the external assignment of a goal.

**Fig. 1:**
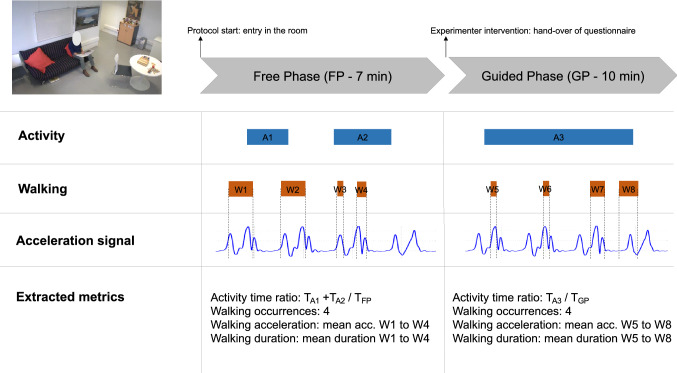
Summarized methodology of extraction of behavioral metrics. A1, A2, and A3 figure an example of a possible distribution across time of activity episodes for a participant, A1 and A2 being activity episodes in the free phase and A3 an activity episode in the guided phase. In this specific case, the computation of activity time ratio is described, for instance for the free phase, as the sum of the durations of A1 and A2 activity episodes divided by the total duration of the free phase. Similarly, W1–W8 figure a possible distribution across time of walking episodes for a participant. In this specific case, walking metrics, for instance for the free phase, are computed as follows: walking occurrences is the total number of walking episodes in the free phase (W1–W4), walking acceleration is the mean acceleration of walking episodes W1–W4, walking duration is the mean duration of walking episodes W1–W4

During the whole scenario, participants’ behavior was video-recorded and their body acceleration was measured using a body sensor (Move II®, Movisens, Karlsruhe, Germany) worn on the hip. Figure 1 summarizes how behavioral metrics were measured for both the free and guided phase (see Supplementary file 1 Part C for further details on behavior coding and extraction of metrics). These behavioral measures extracted from the ECOCAPTURE ecological setting are assumed to be related to apathy defined from a behavioral point of view as a reduction of goal-directed behaviors.

#### Activity time and walking metrics

Relying on previous observations (Batrancourt et al. 2019), we assumed that in the specific ecological situation of the ECOCAPTURE protocol, a reduction of goal-directed behaviors would be manifested in the combination of: (1) a decreased time dedicated to the completion of “overt” goal-directed behaviors and (2) a tendency to wander in the room without being able to initiate and focus on any specific activity. Thus, we were interested in two kinds of behavioral metrics in the free and guided phases: (1) the activity time ratio which directly quantifies the reduction of time spent in goal-directed behaviors and (2) some characteristics of walking episodes (occurrences, acceleration and duration) which quantify the tendency to wander (with frequent, long-lasting walking episodes of low acceleration) and presumably the lack of “goal-directedness” of movements in the room.

From videos, we coded the behaviors of each participant using an ethogram (a list of pre-defined behaviors) and for this study, we were specifically interested in the “activity” and “walking” behaviors. In this study, the “activity” label was attributed to all behavior categories corresponding to sustained series of observable actions, visibly organized towards a coherent purpose (e.g., reading a magazine, playing a game, preparing a drink or any action visibly related to the completion of the questionnaire in the guided phase). The activity time ratio was calculated in the free and guided phases as the ratio of total time spent in sustained goal-directed series of actions (divided by the total duration of the phase). Besides, after coding walking behaviors from videos and extracting the associated acceleration signal, we could calculate the number of occurrences of walking episodes (Walking occurrences), their mean acceleration (Walking acceleration) and their mean duration (Walking duration) for each phase.

### Neuropsychological tests and measures of apathy by questionnaires

To characterize the bvFTD population in terms of disease severity, we used measures of global mental efficiency and cognitive functions: the Mini-Mental State Evaluation (MMSE) (Folstein et al. 1975), the Mattis Dementia Rating Scale (DRS) (Mattis 1976, 1988) and the Frontal Assessment Battery (FAB) (Dubois et al. 2020). To assess the specific syndrome of apathy, we first used the unidimensional Starkstein Apathy Scale (SAS) (Starkstein et al. 1992) (a shortened version of the first scale developed by Marin et al. (1991)) (14 items; e.g., “Do you have motivation?”). Moreover, we used the Dimensional Apathy Scale (DAS) (Radakovic and Abrahams 2014) which consists of three subscales, respectively, measuring the Emotional (8 items; e.g., “I become emotional easily when watching something happy or sad on TV”), Initiation (8 items; e.g., “I set goals for myself”) and Executive (8 items; e.g., “I find it difficult to keep my mind on things”) underlying mechanisms of apathy derived from the theoretical model of apathy subtypes by Levy and Dubois (2006).

### MRI data acquisition and preprocessing

MRI data were acquired at the neuroimaging centre of the Paris Brain Institute with a 3-Tesla Siemens Prisma whole-body scanner with a 12-channel head coil. Anatomical data were T1-weighted images acquired using a magnetization prepared rapid acquisition gradient echo pulse sequence. Resting-state functional data based on the blood oxygenation level-dependent (BOLD) signal were acquired using a T2*-weighted echo-planar image pulse sequence with the following parameters: TR = 2050 ms, TE = 25 ms, flip angle = 80°, acquisition matrix = 68 × 68, FOV = 204 mm. Oblique axial slices of the brain were acquired at 436 time points with a voxel resolution of 3 mm isotropic. Participants were asked to lie with their eyes closed (without falling asleep) during the resting-state acquisition run. MRI data of a total of 34 participants (18 bvFTD and 16 controls) is reported here since the resting-state functional data of two bvFTD patients were either missing or unusable (because of a concomitant vascular disorder potentially impacting the BOLD signal).

MRI data were preprocessed with the default preprocessing pipeline of the CONN toolbox, an open-source Matlab/SPM-based cross-platform software for the analysis of functional connectivity MRI (Whitfield-Gabrieli and Nieto-Castanon 2012). First, functional data were realigned using SPM12 realign and unwarp procedure, where all scans are co-registered and resampled to a reference image (first scan of the first session) using b-spline interpolation. Temporal misalignment between different slices of the functional data was corrected using SPM12 slice-timing correction procedure, where the functional data are time-shifted and resampled using sinc-interpolation to match the time in the middle of each acquisition time. Then potential outlier scans were identified from the observed global BOLD signal and the amount of subject-motion in the scanner. Functional and anatomical data were normalized into standard MNI space and segmented into grey matter, white matter, and CSF tissue classes using SPM12 unified segmentation and normalization procedure. Last, functional data were smoothed using spatial convolution with a Gaussian kernel of 8 mm full width half maximum, in order to increase BOLD signal-to-noise ratio and reduce the influence of residual variability in functional and gyral anatomy across subjects.

### Analyses of behavioral data

The behavioral analyses detailed in the following subsections aimed to: (1) test the behavioral metrics extracted from the ECOCAPTURE setting as potential behavioral markers of apathy; (2) disentangle a dimension characterizing the global reduction of goal-directed behaviors and another dimension corresponding to the specific deficit of self-initiation of goal-directed behaviors. All these analyses were performed using R Studio (Version 1.2.1335).

#### Test of behavioral metrics as potential apathy measures

We first tested the hypothesis that bvFTD patients, characterized by high levels of apathy, would behave differently from HC on the behavioral metrics extracted from the ECOCAPTURE setting. For this purpose, we used a mixed ANOVA design and explored the main and interaction effects of group (between-subjects factor; bvFTD vs HC) and phase (within-subjects factor; free phase vs guided phase) on the raw activity time and walking metrics (extracted in free and guided phases). For all these ANOVA analyses, the normality of dependent variables (in each group × phase condition) was systematically checked and measures identified as extreme outliers (i.e., data points that are more extreme than Q1–3 * IQR or Q3+3 * IQR, where Q1 is the first quartile, Q3 the third quartile and IQR the interquartile range) were removed to better respect the hypothesis of normality. Whenever this hypothesis was not totally satisfied, we further explored the effects of group and phase separately using non-parametric Wilcoxon tests.

We also investigated the Spearman’s rank correlations (across bvFTD patients and controls) between apathy measured by the SAS and the means calculated on the total of free phase and guided phase for activity and walking metrics (e.g., Mean_(FP+GP)_ Activity time ratio). We tested these four correlations as an external validity analysis, to test the hypothesis that the four extracted behavioral metrics may correspond to behavioral markers of apathy. As such, they should correlate with the validated clinical measure of apathy by SAS in the expected direction (negative for activity time ratio and walking acceleration, positive for walking occurrences and duration). Furthermore, we used stepwise regression (i.e., a step-by-step iterative construction of a regression model that involves the selection of independent variables to be used in a final model) to identify the best model predicting SAS measure of apathy from the four behavioral metrics.

#### Dimensional analysis of behavioral metrics and apathy measures by questionnaire

For this dimensional analysis, we used two types of measures: (1) for each of the four behavioral metrics, the calculated means on the total of free and guided phases (e.g., Mean_(FP+GP)_ Activity time ratio) assumed to represent the global reduction of goal-directed behaviors and the calculated differences between free and guided phase (e.g., Delta_(FP-GP)_ Activity time ratio) as potential indicators of the specific (reversible) deficit of self-initiation; (2) the different measures of apathy and apathy subtypes by questionnaires (SAS and DAS subscales). The inclusion of measures by SAS and DAS in this analysis allowed to include measures of apathy that were previously validated in the literature in the extracted dimensions (thus reinforcing their validity as dimensions related to apathy assessment). To disentangle the hypothetical dimensions of global reduction of goal-directed behaviors and specific self-initiation deficit, we used an Exploratory Factor Analysis (EFA) across bvFTD patients and controls, with a promax oblique rotation and a weighted least square estimator. EFA enables the identification of common dimensions underlying the relationships between observed variables. Prior to the extraction of factors, we used the Kaiser–Meyer–Olkin (KMO) Measure of Sampling Adequacy and Bartlett's Test of Sphericity to assess the suitability of data for EFA. The recommended minimum value for the KMO index is 0.6 and the Bartlett's Sphericity Test should be significant at *p* < 0.05 (Tabachnick and Fidell 2007; Hair 2009). We used the scree plot of eigenvalues (Cattell 1966) to investigate the number of factors to extract. To reach a reliable factor solution, we iteratively removed items considered as unrelated to the extracted factors (i.e., with a loading < 0.30 on all factors) (Raubenheimer 2004) and we assessed the internal consistency of the extracted components using Cronbach’s alpha (with a cut-off of 0.60). Individual z-scores on the two extracted factors were automatically calculated using individual data on all measures included in the EFA. To further confirm the validity of dimensions extracted by EFA, we compared the scores on each dimension in bvFTD and controls, assuming that the global reduction of goal-directed behaviors and the specific self-initiation deficit would be higher in bvFTD. We also tested the correlations between extracted dimensions and an objective measure of executive function by the FAB, with the hypothesis that higher reduction of goal-directed behaviors (or higher global apathy) would be related to lower executive function (Nakaaki et al. 2008).

### Analyses of resting-state functional data

All the resting-state functional analyses were performed using the CONN toolbox (version CONN20.b).

#### Fractional amplitude of low-frequency fluctuation

Given the importance of low-frequency fluctuations in determining resting-state activity, the analysis of low-frequency signal power has emerged as a fruitful approach to characterizing the local health of resting-state networks (Zou et al. 2008). The fALFF was calculated for each participant as the voxelwise ratio between low-frequency power (i.e., 0.01–0.1 Hz) and the broader-frequency spectrum of resting-state activity (i.e., 0–0.25 Hz).

#### Seed-based correlation maps

Seed-based connectivity maps represent the level of functional connectivity between a seed and every voxel in the brain. These maps were generated for each participant by calculating the Fisher-transformed bivariate correlation coefficients between each individual voxel BOLD timeseries and the BOLD timeseries averaged over each of seven seed regions of interest. These seeds corresponded to three major hubs of the SN: anterior cingulate cortex (ACC), left and right anterior insula (AI), and four major hubs of the DMN: medial prefrontal cortex (MPFC), posterior cingulate cortex (PCC), left and right lateral parietal cortices (LPC). These selected SN and DMN hubs were assumed to play a key role in the physiopathology of bvFTD (Zhou et al. 2010; Zhou and Seeley 2014) and in the generation of goal-directed behaviors (Raichle et al. 2001). To this end, the seeds provided in CONN were used, which were obtained by independent component analysis of 497 normal subjects from the Human Connectome Project dataset. The MNI coordinates of the centers of mass of the seven selected seeds are reported hereafter: ACC (0, 22, 35), left AI (− 44, 13, 1), right AI (47, 14, 0), MPFC (1, 55, − 3), PCC (1, − 61, 38), left LPC (− 39, − 77, 33) and right LPC (47, − 67, 29).

#### Group-level analyses

The whole-brain fALFF and seed-based connectivity maps of the 7 selected seeds were generated for 18 bvFTD and 16 controls. Then, we tested the respective effects of the two apathy-related dimensions (extracted by EFA) on fALFF and on each seed-based connectivity map, using two separate multiple regression analyses (one for each dimension) within the general linear model framework. Age and sex were included as nuisance covariates in all the tested models. For cluster-level inference, we used a standard criterion for thresholding voxel-based functional activation/connectivity spatial parametric maps while appropriately controlling the family-wise error rate. We used Random Field Theory with a combination of an uncorrected *p* < 0.001 as voxel-level threshold to initially define clusters of interest, and an FDR-corrected *p* < 0.05 (i.e., corrected for false discovery rate) as cluster-level threshold to select among the resulting clusters those deemed significant.

## Results

### Characteristics of participants

The demographic and neuropsychological characteristics of bvFTD patients and healthy controls are described in Table 1. As expected, we found no significant difference between bvFTD and controls for age, sex and education level. BvFTD patients presented significantly lower MMSE, DRS and FAB scores as compared to controls. 15 out of 20 bvFTD patients (75%) were above the Starkstein Apathy Scale pathological cut-off (i.e., SAS $\ge$ 14), while none of the controls were. As expected, SAS score was significantly higher in bvFTD than in controls.

### Investigation of behavioral metrics as objective markers of apathy

#### Effects of group and phase on behavioral metrics

Findings resulting from the ANOVA tests showed that bvFTD patients behaved differently from controls on the extracted activity time and walking metrics.

**Fig. 2:**
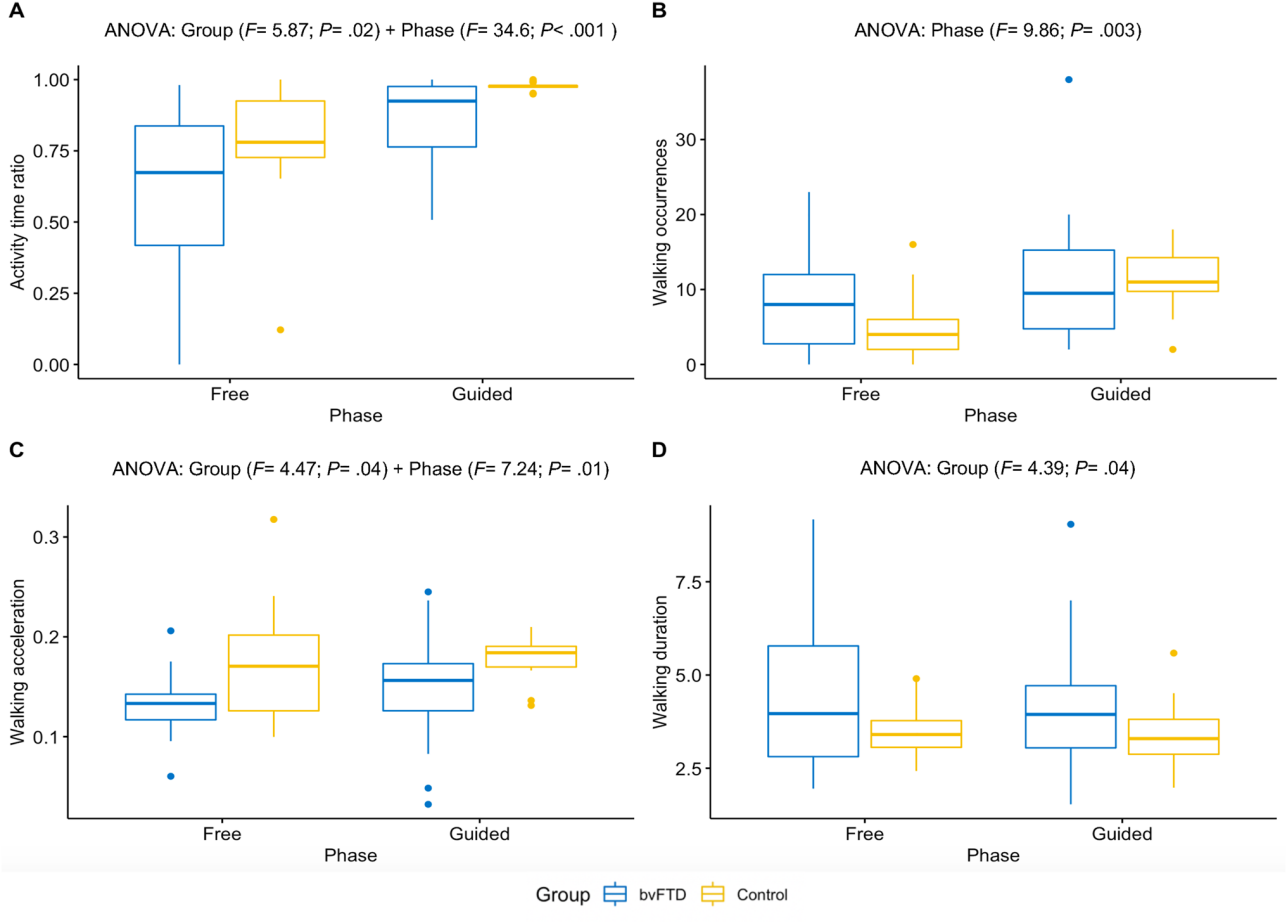
Effects of group and phase on four metrics quantifying goal-directed behaviors. **A** Activity time ratio. **B** Occurrences of walking episodes. **C** Mean acceleration of walking episodes. **D** Mean duration of walking episodes. BvFTD patients: *N* = 20; controls: *N* = 16. Only significant effects obtained from ANOVA tests are displayed for each of the four metrics. Extreme outlier measures were identified and removed: 3 measures of activity time ratio (1 control in free phase/2 controls in guided phase), 2 measures of walking acceleration (1 bvFTD in free phase/1 control in guided phase) and 1 measure of walking duration (1 control in free phase). In the boxplots: horizontal lines represent the first, second (median) and third quartiles of the distribution; vertical bars above and below the boxes figure the lowest 25% and the highest 25% of values, respectively; the dots indicate outliers (which were not identified as extreme outliers)

We found significant main effects of group and phase on the total activity time ratio but no interaction effect (see Fig. 2A). Both bvFTD patients and controls showed increased activity time in guided phase compared to free phase but overall, bvFTD patients showed less activity time than controls. As the hypothesis of normality was not fully satisfied even after removing extreme outlier measures (see all results of normality tests in Supplementary file 2), we confirmed this result by investigating separately the effects of group (*W* = 460; *P* = 0.04) and phase (*W* = 282; *P* < 0.001) on activity time using Wilcoxon tests.

There was only a significant main effect of phase on walking occurrences, the initiation of walking being overall increased in guided phase compared to free phase (see Fig. 2B). This was also confirmed when investigating separately the effects of group (*W* = 700; *P* = 0.50) and phase (*W* = 388; *P* < 0.01) on walking occurrences using Wilcoxon tests. We could not evidence a significant interaction effect from the ANOVA (*F* = 1.78; *P* = 0.19) probably due to lack of statistical power but exploring the effect of phase within each group using Wilcoxon tests revealed that controls presented a significant increase in walking occurrences in guided compared to free phase (*W* = 38; *P* < 0.001), while bvFTD patients did not (*W* = 162; *P* = 0.31). It is noticeable that bvFTD patients tended to show more walking occurrences than controls in the free phase but this difference totally disappeared in the guided phase, as only controls augmented their walking frequency in the guided phase (probably as an adaptive response to the task requiring in-depth exploration of the room in guided phase).

Regarding the acceleration characteristics of walking episodes (in Fig. 2C), we found significant main effects of group and phase. The mean acceleration of walking was higher in guided compared to free phase in both groups and was lower in bvFTD patients compared to controls in both phases.

Finally, we observed only a significant impact of group on walking episode mean duration, with walking episodes lasting longer in bvFTD compared to controls in both phases (see Fig. 2D).

#### Relationship between mean behavioral metrics and SAS

**Fig. 3:**
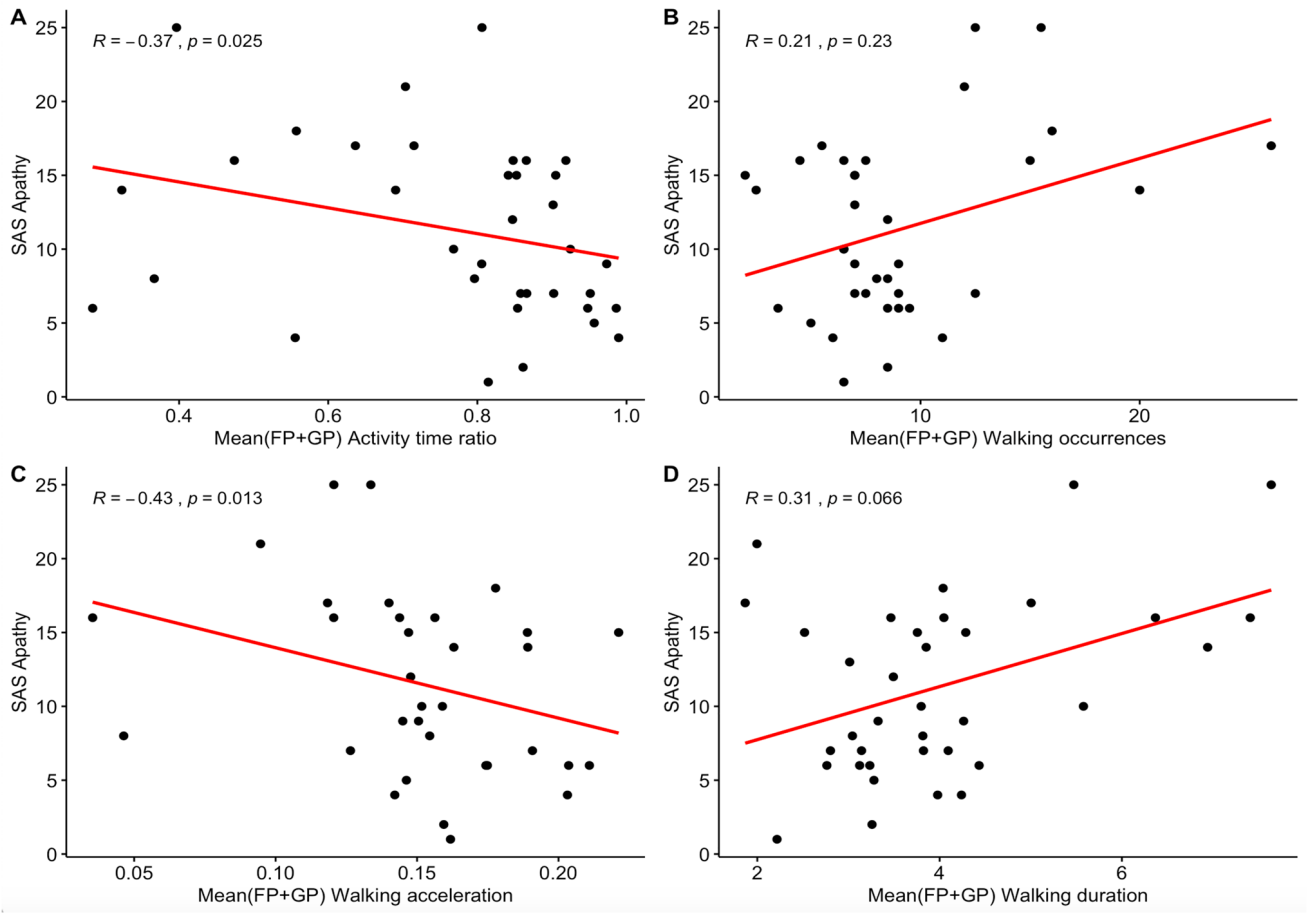
Correlations between apathy measured by the SAS and the four behavioral metrics. **A** Activity time ratio averaged on free and guided phases. **B** Occurrences of walking episodes averaged on free and guided phases. **C** Mean acceleration of walking episodes averaged on free and guided phases. **D** Mean duration of walking episodes averaged on free and guided phases. BvFTD patients: *N* = 20; Controls: *N* = 16. *R* is the Spearman’s rank correlation coefficient between the two variables; SAS, Starkstein Apathy Scale

When investigating Spearman’s correlations between SAS and the averaged behavioral metrics on the total of free and guided phases, we found that all the behavioral metrics evolved with SAS in the expected way (see Fig. 3). When SAS increased, activity time and walking acceleration decreased while walking episode occurrences and mean duration increased. We mostly found medium to large correlations (i.e., between 0.30 and 0.50 in absolute value) with SAS (significant for activity time ratio and walking acceleration, close-to-significant for walking duration). Only the correlation between SAS and walking occurrences was lower than 0.30 (and not significant). However, as observed previously through comparisons between bvFTD and controls, walking occurrences tended to be higher in bvFTD only in the free phase. Relatedly, walking occurrences exclusively in the free phase were more closely correlated to SAS (*R* = 0.43; *P* < 0.01) than walking occurrences averaged on the free and guided phases. Besides, stepwise regression allowed to evidence an optimized model explaining 42% of the variance of apathy measured by SAS, including as predictors: walking occurrences ($\beta$ = 0.43; *P* < 0.01), walking acceleration ($\beta$ = − 0.26; *P* = 0.08) and walking duration ($\beta$ = 0.44; *P* < 0.01).

Therefore, between-group comparisons, correlation and regression analyses are in agreement with the following assumption: in the context of ECOCAPTURE setting, a low time spent in goal-directed behaviors and frequent walking episodes of low acceleration and high duration, are objective behavioral markers of a reduction of goal-directed behaviors. Activity time and walking features can thus contribute to the assessment of apathy.

### Disentangling of two dimensions of apathy

For the EFA applied to the calculated behavioral metrics (means and deltas) and apathy measures by questionnaire, we first calculated the overall KMO index. As it was initially found to be inferior to 0.60, we removed the most “unrelated” item with the lowest KMO (i.e., Delta_(FP-GP)_ Activity time ratio) from the analysis to reach a more acceptable overall KMO > 0.60 (see details in Supplementary file 3 Part A). Bartlett's Test of Sphericity was significant (*P* < 0.05), which further confirmed the suitability of data for EFA. Besides, the scree plot analysis confirmed that a two-factor solution was the best (see details in Supplementary file 3 Part B). We removed another included item: Delta_(FP-GP)_ Walking occurrences, which did not have any loading > 0.30 on the extracted factors (see details in Supplementary file 3 Part C). We thus obtained a well-fitting two-factor structure accounting for 36% of the total variance. Aside from statistical arguments, theoretical considerations justified our choice to remove the deltas calculated for activity time ratio and walking occurrences from the final factor solution shown in Table 2 (see details in Supplementary file 3 Part D).

**Table 2:** Results of the exploratory factor analysis

Observed variables	Factor loadings	
	F1	F2
SAS score	**0.85**	0.27
DAS-Executive score	**0.66**	0.11
Mean_(FP+GP)_ Activity time ratio	**− 0.48**	0.21
Mean_(FP+GP)_ Walking acceleration	**− 0.36**	0.02
Mean_(FP+GP)_ Walking duration	**0.34**	0.12
Mean_(FP+GP)_ Walking occurrences	**0.33**	− 0.05
DAS-Initiation score	0.21	**0.67**
Delta_(FP-GP)_ Walking duration	− 0.12	**0.63**
DAS-Emotional score	0.14	**0.60**
Delta_(FP-GP)_ Walking acceleration	0.12	**− 0.53**

Values indicate the factor loadings of the EFA. Coefficients in bold represent the highest loading (among the two factors) for each item. The calculation of individual scores on F1 and F2 takes into account all the factor loadings onto F1 and F2, respectively. *N* = 36 (bvFTD: *N* = 20/controls: *N* = 16)

Mean_(FP+GP)_, mean calculated on the total of free and guided phases ((FP + GP)/2); Delta_(FP-GP)_, delta calculated as the difference between free and guided phase (FP-GP); activity time ratio, ratio of time spent in goal-directed behaviors; walking occurrences, occurrences of walking episodes; walking acceleration, mean acceleration of walking episodes; walking duration, mean duration of walking episodes; SAS, apathy defined as a global lack of motivation (assessed as a unidimensional construct); DAS-Emotional, diminished integration, processing and expression of emotions; DAS-Initiation, lessened initiation of thoughts and actions; DAS-Executive, inability to manage goals and cognitively strategize to execute a plan of actions

All the means of extracted behavioral metrics loaded on F1 as well as the SAS and DAS-Executive scores. Therefore, F1 was assumed to correspond to the expected dimension of global reduction of goal-directed behaviors. According to the signs of observed loadings, F1 was a pattern characterized by: low time spent in goal-directed behaviors, walking episodes of low acceleration, high frequency and high duration, combined with high self-reported lack of motivation for goal-directed behaviors and high self-reported inability to manage goals.

All the deltas of behavioral metrics loaded on F2 along with DAS-Initiation and DAS-Emotional scores. Thus, F2 was supposed to correspond to the expected dimension assessing the specific deficit of self-initiation of goal-directed behaviors. According to the signs of observed loadings, F2 was a pattern characterized by: higher mean acceleration and lower mean duration of walking episodes in guided phase as compared to free phase, combined with high self-reported lessened initiation of thoughts/behaviors and high self-reported diminished integration and expression of emotions.

Between-group comparisons on extracted scores confirmed that both F1 (*T* = 6.86; *P* < 0.001) and F2 (*T* = 2.37; *P* < 0.05) dimensions were significantly higher in bvFTD compared to controls. Moreover, F1 showed a high negative correlation (*R* = − 0.60; *P* < 0.001) with executive function (i.e., cognitive processes allowing to select and monitor behaviors facilitating the attainment of chosen goals) measured by the FAB, in accordance with the assumption that F1 measures a global reduction of goal-directed behaviors. In contrast, F2 was not related to FAB total score (*R* = − 0.05; *P* = 0.78).

### Resting-state functional connectivity measures and apathy dimensions

#### fALFF and apathy dimensions

**Fig. 4:**
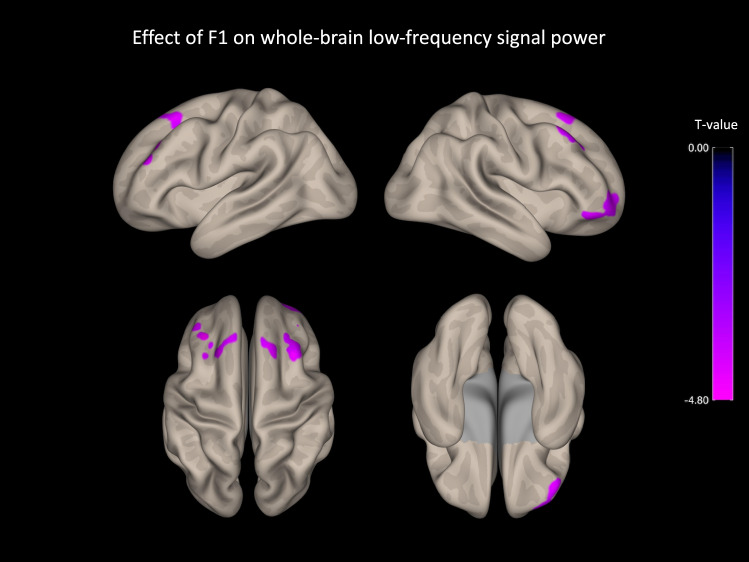
Negative association between F1 and fALFF index of signal power in several regions of the prefrontal cortex. *N* = 34 (bvFTD: *N* = 18/controls: *N* = 16). Effects are corrected for age and sex, and for family-wise error at the level of individual clusters at *P* < 0.05. F1, global reduction of goal-directed behaviors

Figure 4 and Table 3 show the results of group-level analysis regarding the effect of F1 dimension (i.e., global reduction of goal-directed behaviors) on fALFF index. Effect sizes for significant clusters and a detailed analysis of the link between F1 and fALFF within each group (bvFTD and controls) are provided in Supplementary file 4. We did not isolate any significant cluster when exploring the effect of F2 (i.e., specific deficit of self-initiation) on fALFF. Across participants, F1 dimension was negatively related to low-frequency fluctuations in several regions of the prefrontal cortex: the dorsomedial prefrontal cortex (DMPFC), the left and right dorsolateral prefrontal cortex (DLPFC) and the right orbitofrontal cortex (OFC).

**Table 3:** List of significant clusters for group-level analyses on voxelwise low-frequency signal power

Region	*L/R*	Coordinates			Cluster size	*p*-FDR	T-score
		*x*	*y*	*z*			
Effect of F1 on whole-brain fALFF							
Middle frontal gyrus	R	32	22	58	350	< 0.001	− 5.92
Frontal pole	R	46	46	− 20	125	0.02	− 5.25
Frontal pole	R	30	58	− 4	124	0.02	− 4.70
Middle frontal gyrus	L	− 38	34	38	120	0.02	− 5.32
Superior frontal gyrus	L	− 24	26	54	101	0.04	− 5.28

Cluster threshold, *p*-FDR < 0.05; voxel threshold, *p *uncorrected < 0.001; *p*-FDR, FDR-corrected *p* value on cluster size; L/R, left or right hemisphere; fALFF, fractional amplitude of low-frequency fluctuation; N.B., no significant cluster was identified when investigating the effect of F2 on whole-brain fALFF

#### Seed-based connectivity and apathy dimensions

**Fig. 5:**
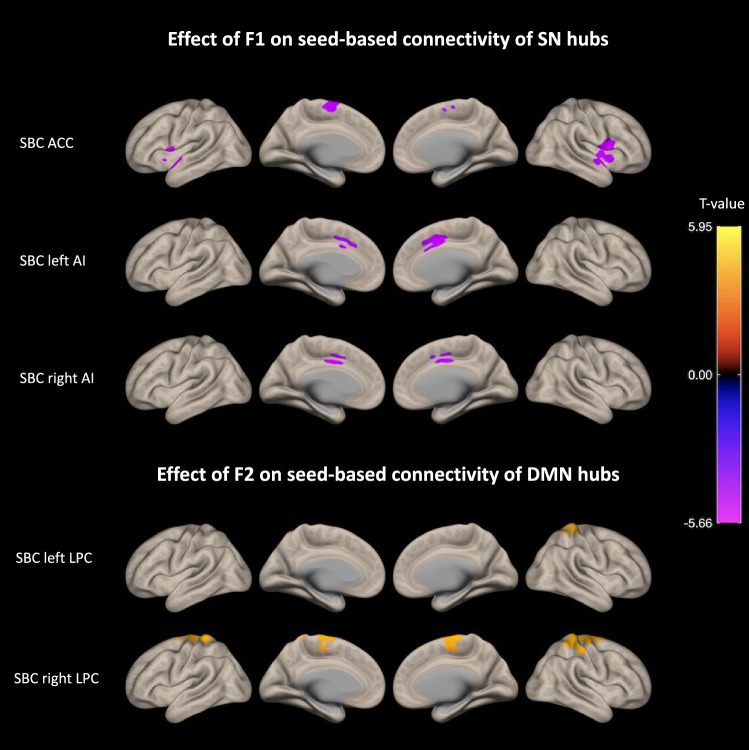
Associations found between F1/F2 and the connectivity of SN/DMN hubs. We observed a negative association between F1 and the seed-based connectivity of SN hubs and on the opposite, a positive association between F2 and the seed-based connectivity of two DMN hubs. *N* = 34 (bvFTD: *N* = 18/controls: *N* = 16). Effects are corrected for age and sex, and for family-wise error at the level of individual clusters at *P* < 0.05. SBC, seed-based connectivity; F1, global reduction of goal-directed behaviors; F2, specific deficit of self-initiation

Figure 5 and Table 4 show the results of group-level analysis regarding the separate effects of F1 and F2 dimensions on the functional connectivity of three seeds of the SN and four seeds of the DMN. Effect sizes for significant clusters and a detailed analysis of the link between F1/F2 and seed-based connectivity within each group (bvFTD and controls) are provided in Supplementary file 4.

**Table 4:** List of significant clusters for group-level analyses on seed-based connectivity maps

Region	L/R	Coordinates			Cluster size	*p*-FDR	T-score
		*x*	*y*	*z*			
Effect of F1 on ACC seed-based connectivity							
Precentral gyrus	R	52	4	14	967	< 0.001	− 5.51
Frontal orbital cortex	R	26	6	− 16	425	0.001	− 6.06
Superior temporal gyrus	L	− 62	0	2	410	0.001	− 5.12
Cerebellum	R	42	− 48	− 32	196	0.03	− 6.03
Accumbens	L	− 14	14	− 8	184	0.03	− 4.83
Superior frontal gyrus	L	− 6	2	74	172	0.04	− 4.38
Effect of F1 on left AI seed-based connectivity							
Anterior cingulate cortex	R	6	6	46	528	< 0.001	− 4.98
Cerebellum	R	16	− 84	− 44	278	0.008	5.35
Cerebellum	L	− 16	− 84	− 42	271	0.008	5.88
Effect of F1 on right AI seed-based connectivity							
Cerebellum	R	10	80	− 40	548	< 0.001	6.57
Anterior cingulate cortex	L	− 2	− 2	40	279	0.008	− 4.85
Effect of F2 on right LPC seed-based connectivity							
Post-central gyrus	R	24	− 30	70	1322	< 0.001	6.83
Post-central gyrus	L	− 22	− 32	68	424	< 0.001	5.67
Effect of F2 on left LPC seed-based connectivity							
Post-central gyrus	R	22	− 32	68	408	0.001	5.42
Brain Stem	L	− 2	− 32	− 4	348	0.001	− 7.66

Cluster threshold, *p*-FDR < 0.05; voxel threshold, *p *uncorrected < 0.001; *p*-FDR, FDR-corrected *p* value on cluster size; L/R, left or right hemisphere; ACC, anterior cingulate cortex; AI, anterior insula; LPC, lateral parietal cortex

We found that, across participants, F1 (assessing the global reduction of goal-directed behaviors) was associated with a decreased connectivity between the three SN hubs (i.e., from ACC seed to left/right frontoinsular regions and from left/right AI seeds to ACC), a decreased connectivity between the ACC and the ventral striatum, and a decreased connectivity between the ACC and the dorsomedial frontal cortex (i.e., supplementary motor area—SMA). Although not shown on Fig. 4, F1 was also related to increased connectivity between left/right AI and the cerebellum (medial part). F1 was not related to the connectivity of DMN hubs. Exploring the relationship between F1 and the connectivity of SN hubs within each group (bvFTD and control) led us to observe that previously described effects were mostly driven by the bvFTD group.

On the opposite, F2 (assessing the specific deficit of self-initiation) was linked with an increased connectivity of two DMN hubs (left and right LPC) and was not associated with the connectivity of SN hubs. Indeed, F2 was found to increase with the connectivity between bilateral LPC and medial frontal/parietal cortex. In particular, F2 was associated with an increased connectivity between the right LPC and the medial part of superior frontal gyrus, pre- and postcentral gyri (including SMA). Of note, F2 was also related to decreased connectivity between left LPC and a brain stem region. The links between F2 and the connectivity of DMN hubs were driven by both the bvFTD and control group and remained significant after controlling for the clinical status.

## Discussion

The present investigation on apathy in bvFTD, based on multi-modal assessments including an ecological approach of behavior tracking, allowed us: (1) to identify objective behavioral measures contributing to the assessment of apathy; (2) to disentangle two dimensions describing the global severity of apathy and the specific contribution of a self-initiation deficit; (3) to evidence that global apathy severity and self-initiation deficit were associated with two distinct patterns of modified functional connectivity.

### Objective behavioral markers of apathy

We found that bvFTD patients (known to present high levels of apathy) spent less time in goal-directed behaviors and presented less “goal-directed” walking episodes (of lower acceleration and higher duration) than controls did. Both bvFTD patients and controls similarly increased their time spent in goal-directed behaviors and the acceleration of walking in the guided phase compared to the free phase. This last result suggested that like controls, bvFTD patients could benefit from external guidance to quantitatively increase goal-directed behaviors. However, no group-phase interaction was evidenced and the discrepancy between bvFTD and controls was maintained in guided phase, which indicates that external guidance cannot totally compensate for the deficit of goal-directed behaviors in bvFTD.

As confirmed by correlation analyses, using an ecological approach allowed us to put forward original behavioral markers of apathy in bvFTD patients, in particular three characteristics of walking episodes (high frequency, low acceleration and high duration) which were found to be the best predictors of apathy measured by clinical scale (SAS). Like the measure of apathy by SAS, these walking features (markers of wandering in the room in the specific context of ECOCAPTURE protocol) may, therefore, highly contribute to capture the lack of motivation/interest in goal-directed behaviors (either with or without external drive). These results relating walking features to apathy suggest that the goals directing participants’ actions may unconsciously impact the way their body moves in the room. Changes in motor activity have been suggested as markers of apathy for a long time but the link between walking characteristics and apathy has never been demonstrated with this level of precision in an ecological paradigm. One study (Groeneweg-Koolhoven et al. 2015) had already suggested a relationship between apathy and walking speed but it was not based on an ecological approach. They investigated the correlates of apathy (assessed by Marin’s Apathy Evaluation Scale) in non-demented older individuals, either depressed or non-depressed. In depressed older individuals, walking speed (determined by measuring the time needed to complete a six-meter walk) was found to be slower in those with apathy compared to those without apathy. Within the domain of ecological approaches, two studies of ambulatory actigraphy (i.e., continuous acceleration measures for several days thanks to a wrist actigraph) in patients with Alzheimer’s disease showed that the patients’ apathy scores on neuropsychiatric assessment scales correlated negatively with the mean motor activity (i.e., the mean number of activity counts per minute extracted from the recorded acceleration signal) (David et al. 2010, 2012). Our findings emphasize this relationship further: indeed, we show that not only is the long-term mean motor activity (derived from the acceleration signal) related to apathy but also the mean acceleration exclusively, while walking in a room on a short-term period.

### Two dimensions of apathy

Calculated means and deltas for the activity time and walking metrics, along with measures of apathy by clinical scales, contributed to extract two dimensions: a first dimension (F1, related to the means of behavioral metrics) assumed to characterize the global reduction of goal-directed behaviors and a second dimension (F2, related to the deltas of behavioral metrics) supposed to represent the specific deficit of self-initiation.

The first extracted dimension corresponded to a behavioral pattern characterized by a low time spent in goal-directed behaviors and a general tendency to wander in the room. It was also related to self-reported lack of motivation (measured by SAS) and to a self-reported difficulty to implement a plan of actions towards goal management (measured by DAS-Executive). This finding indicates that, contrary to a common belief, the reality of apathy syndrome is not limited to standing still and doing absolutely nothing. On the opposite, it can include stereotyped aimless movements, like observed in the most severe cases of apathy in patients with an auto-activation deficit syndrome (Laplane and Dubois 2001), which may precisely prevent from focusing on goal management.

The second extracted dimension corresponded to a pattern of enhanced ‘goal-directedness’ of walking when the initiation of goal-directed behaviors was facilitated by external guidance. Thus, typically, patients with high scores on this second dimension would have specific difficulties in self-initiating goal-directed behaviors but might be able to complete them with external guidance. This dimension was also associated with the self-reported impairment of emotional-affective and auto-activation processing (DAS-Emotional and DAS-Initiation). The emotional-affective aspect of apathy has been shown to be very salient in bvFTD (Kumfor et al. 2018; Radakovic et al. 2021), which may explain that this mechanism contributes to the specific deficit of self-initiation of goal-directed behaviors, along with the difficulty to initiate thoughts and actions. Executive functions (DAS-Executive and FAB) were not related to the dimension of self-initiation deficit, suggesting that this measure of self-initiation deficit was independent of the cognitive strategizing for the management of goals.

### Functional connectivity related to the two dimensions of apathy

The F1 dimension of reduction of goal-directed behaviors (or global apathy) was related to lower low-frequency signal power within several distinct regions of the PFC: the right OFC, the bilateral DLPFC and DMPFC. These regions correspond to the frontal part of the three frontostriatal circuits associated with apathy in the model proposed by Levy and colleagues (Levy and Dubois 2006; Levy 2012; Godefroy et al. 2020). This model was partly supported by empirical evidence using structural imaging. Indeed, Massimo and colleagues (Massimo et al. 2015) assessed three components of goal-directed behaviors (i.e., motivation, planning, and initiation) in bvFTD patients using the Philadelphia Apathy Computerized Test and they found that poor motivation was related to grey matter atrophy in OFC, planning impairment to atrophy in DLPFC, and poor initiation to atrophy in ACC.

Interestingly, there was no overlap between the brain regions for which local resting state activity was related to global apathy (i.e., OFC, DLPFC and DMPFC) and the brain regions for which distant connectivity was associated with global apathy (i.e., mostly ACC, left/right AI, ventral striatum and SMA). The investigation of local resting state activity in association with apathy evidenced PFC areas of which grey matter atrophy was consistently related to apathy (or apathy subtypes) in previous literature regarding FTD symptoms (Ducharme et al. 2018). On the opposite, the analysis of distant connectivity from several seeds revealed the involvement of regions that were less typically associated with apathy by structural neuroimaging in FTD, like the AI or the SMA (Ducharme et al. 2018), or even regions found to be associated with apathy on isolated occasions like the cerebellum (Shaw et al. 2021) or the brain stem (ventral tegmental area) (Schroeter et al. 2011). Therefore, the global reduction of goal-directed behaviors may arise from the complex combination of: (1) local changes in connectivity in close association with local grey matter atrophy and (2) global changes in connectivity between more distant regions of the brain that are less directly related to grey matter atrophy.

Through the analysis of the distant functional connectivity between selected hubs of the SN/DMN and the rest of the brain, we identified two distinct patterns of modified connectivity relating to the two dimensions of apathy. A decreased connectivity between SN hubs (AAC and bilateral AI) correlated with global apathy, while an increased connectivity of DMN hubs (bilateral LPC) with dorsomedial regions correlated with the specific deficit of self-initiation. These findings were in agreement with the model of connectivity changes in bvFTD suggested by Zhou, Seeley and colleagues (Zhou et al. 2010; Zhou and Seeley 2014). These authors had shown that global clinical severity in bvFTD correlated with loss of right frontoinsular SN connectivity and enhancement of parietal DMN connectivity, suggesting that functional connectivity reductions and enhancements both carried the potential to track the progression of disease-related disorders (Zhou et al. 2010). Besides, two previous studies of MRI resting state functional connectivity in bvFTD also found links between apathy (assessed as a unidimensional construct with the Frontal Behavioral Inventory) and the connectivity within SN (Day et al. 2013; Farb et al. 2013) and within DMN (Farb et al. 2013). An additional study (Zhou et al. 2020) applied a graph theoretical analysis to analyze the topological properties of cerebral blood flow network based on single photon emission tomography in bvFTD patients with and without apathy. They observed that, compared with bvFTD patients without apathy, patients with apathy exhibited additional loss of hubs in regions commonly associated with the SN (including ACC, AI, ventral rostral PFC areas, and subcortical regions), but recruited more hubs in areas belonging to the DMN (including angular gyrus, precuneus and posterior cingulate cortex).

Results suggested that the connectivity of ACC plays a key role in the quantitative production (both initiation and maintenance) of goal-directed behaviours. As predicted by Zhou and Seeley’s working functional–anatomic model of the SN, the functional connectivity between the ACC and AI (or frontoinsula—FI) was involved in the severity of global apathy syndrome (assessed by F1). According to this model, the ACC node coordinates with the AI, which processes the major ascending input streams regarding the moment-to-moment condition of the body to elaborate ‘feeling state’ representations. According to the ‘feeling state’ communicated by the AI, the ACC can mobilize adapted behavioural responses through: (1) the recruitment of executive and task control network resources; (2) the inhibition of the DMN to keep attention focused on the ongoing task. Previous studies also found a relationship between the connectivity of dorsal ACC and apathy in patients with AD (Tumati et al. 2020), in patients with late-life depression (Yuen et al. 2014) and in patients with Parkinson’s disease (Sun et al. 2020). Besides, the functional connectivity between dorsal ACC and SMA had been shown to decrease with apathy as a trait in healthy subjects (Bonnelle et al. 2016), which was replicated in our study in a sample mixing bvFTD patients and healthy controls.

Results also evidenced an important role of increased connectivity between parietal DMN hubs (in particular the right LPC) and dorsomedial regions of motor and pre-motor cortices in the specific ability to self-initiate goal-directed behaviors. The activation of the DMN is generally recognized to be responsible for an introspective state which prevents the allocation of cognitive resources on an extrinsic goal-directed task (Mak et al. 2017). An increased connectivity of the DMN may reduce the capacity to intrinsically deactivate this network and thus disturb the self-initiation of goal-directed behaviors. More specifically, the involvement of LPC is in line with a recent review of literature on the association between LPC dysfunction and apathy across disorders (Tumati et al. 2019). Considering the evidence, Tumati and colleagues suggested a revised model of apathy in which impaired internal initiation of behavior mediated by a dysfunction of the lateral parietal lobule may be sufficient (although not necessary) to reduce goal-directed behavior, and would constitute a volitional subtype of apathy. More precisely, the authors proposed that the neural processes in the lateral parietal lobule may contribute to transform internal goals into external actions through a process of embedding intended actions in a ‘body schema’ which facilitates the adequate recruitment of an effector system. An increased connectivity between the LPC and medial motor/pre-motor regions may thus: (1) reflect an inadequate recruitment of the effector system by the LPC; (2) constitute a compensatory mechanism for a disturbed embedding of intended actions in a ‘body schema’.

Finally, results suggested the importance of SMA as an efferent motor system involved in both the global apathy and the specific self-initiation deficit. In bvFTD patients, the global reduction of goal-directed behaviours might be related to the decreased connectivity of ACC with afferent salience nodes (i.e., AI) and efferent motor nodes (i.e., SMA). In some patients with relatively preserved connectivity of ACC, LPC dysfunctions combined with an enhanced connectivity between LPC and SMA might be sufficient to prevent the spontaneous self-initiation of goal-directed behaviors without suppressing the capacity to generate these behaviors with external guidance. This is consistent with the assumption that dorsomedial frontal areas are linked to the initiation component of apathy (Ducharme et al. 2018). In general, the pre-SMA/SMA region, which integrates inputs from the PFC and basal ganglia, is assumed to play a major part in the initiation of voluntary action (Haggard 2008).

### Methodological limits

The relatively small sample size of bvFTD patients is a first limitation of the present study. The results need to be replicated in other larger samples. Small sample size is a common issue in studies investigating the neural bases of apathy in FTD (Ducharme et al. 2018). This is due to the heavy requirements of our protocol (2 days of experimental protocol with extensive neuropsychological testing) and to our selective inclusion criteria for bvFTD patients (e.g., MMSE score > 20).

A second potential limitation is that the objective assessment of apathy is only at the behavioral level (via the activity and walking behavioral metrics), but not in other dissociable domains of apathy mechanisms (cognitive and emotional) (Robert et al. 2009). Objective measures of apathy in ecological context (until now, mostly physiological measures of motor activity by actigraphy) only exist at the behavioral level. In this study, we used the validated subjective measures of apathy dimensions by the DAS questionnaire, because this allowed to include the emotional, initiation and executive aspects of apathy in the extracted dimensions (global apathy and self-initiation deficit) and thus to confirm the specific mechanisms involved in each type of deficit (i.e., executive mechanisms mostly involved in global apathy/emotional and initiation mechanisms mostly involved in self-initiation deficit). Besides, although one of our goal was to identify objective and ecological behavioral markers associated with apathy, we do not deny that the subjective aspect of apathy (measured by the SAS and DAS in our study) is also an important part of the syndrome, complementary to the objective aspect. However, these subjective self-report measures were potentially affected by anosognosia in some bvFTD patients (Levy 2012).

The extent of the impact of anosognosia is difficult to estimate, because we lack reports from the caregiver. Different arguments support a limited impact of anosognosia on the results of the factor analysis. First, both objective and subjective measures loaded on the two extracted dimensions (they were not divided into one dimension of objective measures and one dimension of subjective measures). Therefore, despite the effect of anosognosia, objective and subjective measures were still explained by a same latent concept (assumed to be global apathy for measures loading on F1 and specific self-initiation deficit for measures loading on F2). Second, dimensions were extracted by factor analysis across the total sample of bvFTD patients and controls and anosognosia could only affect bvFTD patients. Finally, the validity of the global apathy dimension was confirmed by its high inverse correlation with an objective measure of executive function. The main potential impact of anosognosia is, therefore, the under-estimation of global apathy and self-initiation deficit in some bvFTD patients presenting high anosognosia. However, we are confident that taking account of the potential impact of anosognosia on extracted dimensions of apathy would not change the observed effects on connectivity. Indeed, for all the detected effects, a similar trend (i.e., decrease in connectivity of SN hubs with higher global apathy and increase in connectivity of parietal DMN hubs with higher self-initiation deficit) was observed both within bvFTD patients and within controls, who are not affected by anosognosia.

Further minor measurement biases need to be reported. First, the measure of time spent in goal-directed behaviors (i.e., activity time ratio metric) may have been partly biased by the subjective interpretation of the objective underlying subject’s observable behavior by the examiners in charge of the coding of behaviors (especially in the free phase). Nonetheless, the excellent level of interrater reliability (see Supplementary file 1) suggests that this subjective bias was very limited. Besides, as language deficits might occur in bvFTD patients (Geraudie et al. 2021), in the absence of objective measures of oral/written comprehension to complete the clinician’s perception that the patient was able to understand all the performed tasks/questionnaires (defined as an inclusion criteria), we cannot totally exclude that this type of difficulties might have slightly biased behavior and answers to questionnaires in some patients with higher cognitive impairments. However, comprehension biases were probably limited by: (1) the fact that included bvFTD patients were at early stages of the disease (all MMSE scores > 20); (2) the absence of complex instructions during the close-to-real life situation (even in the guided phase) in which behavioral data were collected; (3) the supervision of the filling of questionnaires by an examiner (a neuropsychologist) to ensure that questionnaires were correctly understood.

## Conclusions

Our study highlights that along with the time spent in activity, walking characteristics under close-to-real-life situations could be very useful in complementing subjective measures of apathy, thus leading to a more thorough and precise quantification of apathy. This assessment approach combining objective and subjective measures could be extended to a broad range of behavioral symptoms and could thus prove useful for a better understanding of the relationship between brain connectivity and behavior.

Using the lesion model of patients with bvFTD, our results provided the first empirical evidence of several aspects of Tumati and colleagues’ theoretical framework of goal-directed behavior, ascribing cognitive control of voluntary motor behavior to the lateral parietal cortex (Tumati et al. 2019). According to this framework, an intention to act is generated in the lateral parietal cortex, which triggers preparation for movement. The lateral parietal cortex selects effectors of action and initiates action by sending excitatory impulses to the motor cortex. In parallel, the lateral parietal cortex connects with the dorsal ACC to assess various alternative paths to the goal and select the appropriate goal and path. Moreover, according to Tumati’s model, SMA would play a central role in the pathways through which motor behavior is regulated by the lateral parietal cortex and dorsal ACC. Our observations confirm that: (1) globally reduced goal-directed behaviors (due to impaired executive functions) are related to impaired connectivity between ACC and SMA; (2) a specific deficit of self-initiation (due to poor control of voluntary motor initiation) is associated with modified connectivity between lateral parietal cortex and medial motor and pre-motor regions (including SMA). In particular, we suggest that the enhanced connectivity between lateral parietal cortex and midline motor regions might serve as a biomarker of reduced volition even among healthy subjects. Further investigation into the longitudinal evolution of functional connectivity leading to apathy and to self-initiation deficit in bvFTD could provide further insight into the dynamic causal relationships between connectivity changes and the production of voluntary goal-directed behaviors.

## Supplementary Information

Below is the link to the electronic supplementary material.

Supplementary file1 (PDF 122 kb)

Supplementary file2 (PDF 53 kb)

Supplementary file3 (PDF 175 kb)

Supplementary file4 (PDF 18066 kb)

## Data Availability

The data that support the findings of this study are available from the corresponding author, upon reasonable request.

## Abbreviations

ACC: Anterior cingulate cortex
AI: Anterior insula
bvFTD: Behavioural variant frontotemporal dementia
DAS: Dimensional Apathy Scale
DLPFC: Dorsolateral prefrontal cortex
DMN: Default mode network
DRS: Mattis Dementia Rating Scale
EFA: Exploratory Factor Analysis
FAB: Frontal Assessment Battery
fALFF: Fractional amplitude of low-frequency fluctuations
FTD: Frontotemporal dementia
LPC: Lateral parietal cortex
MCC: Midcingulate cortex
MMSE: Mini-mental state evaluation
MPFC: Medial prefrontal cortex
OFC: Orbitofrontal cortex
PCC: Posterior cingulate cortex
PFC: Prefrontal cortex
ROI: Region of interest
SAS: Starkstein Apathy Scale
SFG: Superior frontal gyrus
SMA: Supplementary motor area
SN: Salience network
VBM: Voxel-based morphometry
