# Supplementary material for: Functional connectivity correlates of reduced goal-directed behaviors in behavioural variant frontotemporal dementia
Source: Brain Struct Funct. 2022 Jun 25;227(9):2971–89. doi: 10.1007/s00429-022-02519-5 (PMC9653340; doi:10.1007/s00429-022-02519-5)
Supplement: Supplementary file 1 — Supplementary file1 (PDF 122 kb) [file 429_2022_2519_MOESM1_ESM.pdf]

Supplementary file 1

A/ ECOCAPTURE protocol overview

The recruitment period of the ECOCAPTURE protocol started in September 2017. Within the ECOCAPTURE protocol, an ecological setting was used to observe and record participants' behaviour during a predetermined 45-minute multiple-phase script (described in section B). This setting reproduced a close-to-real-life situation in a functional exploration platform (PRISME, ICM core facility, Salpêtrière hospital, Paris, France) transformed into a fully furnished waiting room and equipped with video and a sensor-based data acquisition system (including an accelerometer) that tracked the participant's behaviour. Participants were told that they had to wait in a room that serves as a staff lounge before doing further tests and they were left alone in the room (except during a few interventions of the experimenter between the phases of the script). At the time of initial consent, participants had been informed that their behaviour would be recorded by video cameras located in the room. Apart from undergoing the 45-minute scenario for ecological behavioural recording, all participants of the ECOCAPTURE protocol carried out extensive neuropsychological assessments and a brain MRI protocol.

B/ Description of the ECOCAPTURE scenario

The ECOCAPTURE paradigm mimics a naturalistic situation (i.e., waiting time in a comfortable waiting room) with a structured scenario designed to obtain objective measures of behavioural syndromes such as apathy or disinhibition.

Prior to the experimental session, the examiner asked the participant (and/or caregiver) to indicate his/her preferences for food, drinks, types of magazines, and music. The room and the scenario (type of music displayed as positive environmental stimulation) were customized accordingly.

Outside of the room (in the adjacent monitoring room), the examiner told the participant that he/she was going to wait a little in a staff lounge before doing more tests. The participant was then equipped with the device used to record the acceleration signal: a ®Movisens triaxial accelerometer worn on the hip during the whole ECOCAPTURE scenario.

As described below, the ECOCAPTURE scenario lasted for a total of about 45 minutes and was divided into 5 phases interrupted by short examiner interventions.

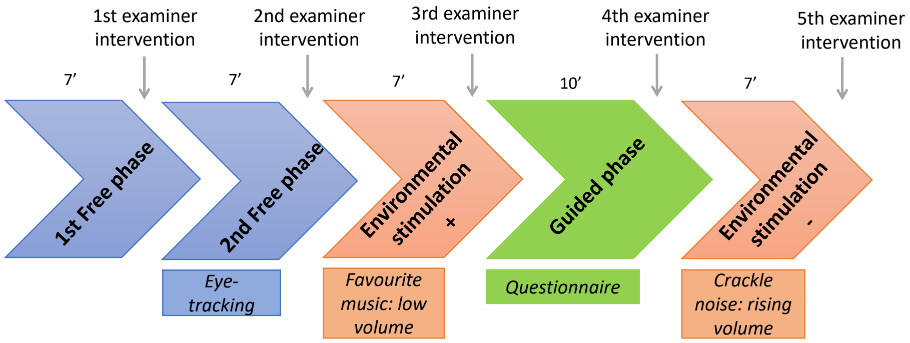

For our research, we focused specifically on the first free phase and on the guided phase detailed hereafter.

<u>Details on the first free phase (FP-7 minutes)</u>

The examiner invited the participant to enter the room, to make himself/herself comfortable in the room. Video coding of behaviours for FP was started when the observer left the room, leaving the participant alone in the waiting room.

This first phase was self-guided: subjects were simply invited to make themselves at ease in the room and to use the provided objects at their own convenience. They could use the space (e.g., sit on a chair around a table or on a sofa) and use provided foods, drinks, magazines, books, games, etc… For instance, some subjects used the kettle to boil water and prepared a cup of coffee or tea.

<u>Details on the guided phase (GP-10 minutes)</u>

After knocking the door, the examiner entered and brought the questionnaire to the participant, asking him/her to fill it out. The subject often asked for pens. The examiner simply said that everything needed to fill the questionnaire was in the room and no

indication was given about the location of pens. Video coding of behaviours for GP was started when the participant was once again alone in the waiting room.
The questionnaire handled by the examiner was divided into five parts and the first written instruction was to complete the questionnaire in the following order and with the required pen:

QUESTIONNAIRES	PEN
QUESTIONNAIRE « FOOD ITEMS IN THE ROOM »	Blue pen
QUESTIONNAIRE « DEMOGRAPHIC »	Black pen
QUESTIONNAIRE « SLEEP »	Blue pen
QUESTIONNAIRE « MEALS »	Green pen
QUESTIONNAIRE « FURNITURE IN THE ROOM »	Red pen

All questionnaires contained very easy questions: either personal questions on age, job, marital status, height, weight, quality of sleep, preferences for meals… or questions requiring exploration of the room like selecting food items present in the room from a series of pictures of foods.
The most challenging aspect of the questionnaire was to fill it out with the correct pens. For instance, one part of the questionnaire had to be filled using a green pen. Finding the green pen in the room implied firstly, to find the box in which it was hidden, and secondly, to find the key to open the box.

C/ Extraction of the behavioural metrics from FP and GP

Using the six videos covering the different viewpoints of the waiting room as well as the ethogram resource, behaviours were coded for the FP and GP using the ECOCAPTURE ethogram. This ethogram included the following categories of behaviour on which we focused: “activity”, “questionnaire-related activity”,

“questionnaire-related exploration” (for the calculation of goal-directed activity time ratio in FP and GP) and “walking” (for the calculation of walking episode characteristics in FP and GP). In the FP, activity time ratio included only episodes labelled as “activity” whereas in the GP, it included also episodes labelled as “questionnaire-related activity” and “questionnaire-related exploration”.

Definitions of the behavioural categories selected from the ethogram:

- **Activity**:
  Visibly coherent purposeful actions, sustained for more than 10 seconds (e.g., reading, eating, preparing a drink, playing games)
    - **Questionnaire-related activity (only in guided phase)**:
      Coherent purposeful actions visibly contributing to the filling of the questionnaire in the GP (e.g., reading the questionnaire, writing answers to the questionnaire, weighting with the provided scale)
    - **Questionnaire-related exploration (only in guided phase)**:
      Explorations of the room visibly contributing to the filling of the questionnaire in the GP (e.g., opening the drawers of a cupboard to find the pens or to find the key to open a box containing a pen).
- **Walking**:
  Walking episodes are all the moments when the participant performs more than two steps to move in the room. Trampling is not included in walking episodes.

The video-based behaviour coding was generated by using a manual video annotation tool (The Observer XT®, Noldus, Wageningen, The Netherlands). Inter-coder reliability was calculated in a subsample representing more than 20% of the participants (n = 8; 4 bvFTD and 4 HC). For this subsample, two different examiners coded the videos; one of them was blinded to the group identities of the subjects. All calculated Cohen’s kappa coefficients were superior to 0.98, indicating close-to-perfect agreement between raters and therefore excellent interrater reliability.

We used the values of movement acceleration (in g) automatically extracted at each second by the ®Movisens DataAnalyzer application. Through a saturation of the acceleration signal at the end of the ECOCAPTURE protocol (intense movement made

by the experimenter with the accelerometer), we were able to calculate, for each participant, the offset between video and acceleration signal and thus to match coded behaviours with the corresponding acceleration signal.
