# Supplementary material for: Functional connectivity correlates of reduced goal-directed behaviors in behavioural variant frontotemporal dementia
Source: Brain Struct Funct. 2022 Jun 25;227(9):2971–89. doi: 10.1007/s00429-022-02519-5 (PMC9653340; doi:10.1007/s00429-022-02519-5)
Supplement: Supplementary file 2 — Supplementary file2 (PDF 53 kb) [file 429_2022_2519_MOESM2_ESM.pdf]

Supplementary file 2

Results of Shapiro normality tests for ANOVA testing the effects of group and phase on behavioral metrics

A/ Normality tests on *Activity time ratio* in each group x phase condition

- Before removing 3 extreme outliers

Group	Phase	Statistic (W)	*p*-value
bvFTD patients	Free	.94	.22
healthy controls	Free	.77	.001
bvFTD patients	Guided	.83	.003
healthy controls	Guided	.51	< .001

- After removing 3 extreme outliers

Group	Phase	Statistic (W)	*p*-value
bvFTD patients	Free	.94	.22
healthy controls	Free	.79	.003
bvFTD patients	Guided	.83	.003
healthy controls	Guided	.90	.10

B/ Normality tests on *Walking occurrences* in each group x phase condition

(No detected extreme outliers)

Group	Phase	Statistic (W)	*p*-value
bvFTD patients	Free	.90	.05
healthy controls	Free	.86	.02
bvFTD patients	Guided	.86	.007
healthy controls	Guided	.97	.836

C/ Normality tests on *Walking acceleration* in each group x phase condition

- Before removing 2 extreme outliers

Group	Phase	Statistic (W)	*p*-value
bvFTD patients	Free	.93	.16
healthy controls	Free	.92	.22
bvFTD patients	Guided	.96	.51
healthy controls	Guided	.75	.001

- After removing 2 extreme outliers

Group	Phase	Statistic (W)	*p*-value
bvFTD patients	Free	.96	.62
healthy controls	Free	.92	.23
bvFTD patients	Guided	.96	.51
healthy controls	Guided	.92	.26

D/ Normality tests on *Walking duration* in each group x phase condition

- Before removing 1 extreme outlier

Group	Phase	Statistic (W)	*p*-value
bvFTD patients	Free	.93	.18
healthy controls	Free	.84	.01
bvFTD patients	Guided	.93	.16
healthy controls	Guided	.96	.64

- After removing 1 extreme outlier

Group	Phase	Statistic (W)	*p*-value
bvFTD patients	Free	.93	.18
healthy controls	Free	.93	.30
bvFTD patients	Guided	.93	.16
healthy controls	Guided	.96	.64
