# Supplementary material for: Functional connectivity correlates of reduced goal-directed behaviors in behavioural variant frontotemporal dementia
Source: Brain Struct Funct. 2022 Jun 25;227(9):2971–89. doi: 10.1007/s00429-022-02519-5 (PMC9653340; doi:10.1007/s00429-022-02519-5)
Supplement: Supplementary file 3 — Supplementary file3 (PDF 175 kb) [file 429_2022_2519_MOESM3_ESM.pdf]

Supplementary file 3

Detailed reasoning towards an optimized factor structure by exploratory factor analysis (EFA)

A/ Reaching a suitable Kaiser-Meyer-Olkin index of factor adequacy

The Kaiser-Meyer-Olkin (KMO) Test is a measure of how suited the data is for factor analysis. The statistic is a measure of the proportion of variance among variables that might be common variance. The higher the proportion, the higher the KMO-value, the more suited the data is to factor analysis. KMO values less than 0.6 indicate the sampling is not adequate and that remedial action should be taken.

When computing the initial KMO with all 12 items (8 behavioral metrics and 4 measures by questionnaire) included in the EFA, we found that the overall KMO was only 0.55. Thus we explored the measure of sampling adequacy (MSA - indicates to what extent an item is suitable for a factor analysis) for each of the 12 items (see table below). After removing the item with the lowest MSA (i.e., $Delta_{(FP-GP)}$ Activity time ratio), we reached a more suitable overall KMO of 0.63.

$Mean_{(FP+GP)}$ Activity time ratio	$Delta_{(FP-GP)}$ Activity time ratio	$Mean_{(FP+GP)}$ Walking occurrences	$Delta_{(FP-GP)}$ Walking occurrences	$Mean_{(FP+GP)}$ Walking acceleration	$Delta_{(FP-GP)}$ Walking acceleration	$Mean_{(FP+GP)}$ Walking duration	$Delta_{(FP-GP)}$ Walking duration
0.42	0.35	0.51	0.43	0.45	0.64	0.49	0.54

SAS score	DAS-Emotional score	DAS-Initiation score	DAS-Executive score
0.71	0.64	0.67	0.69

B/ Scree plot analysis to determine the optimal number of factors

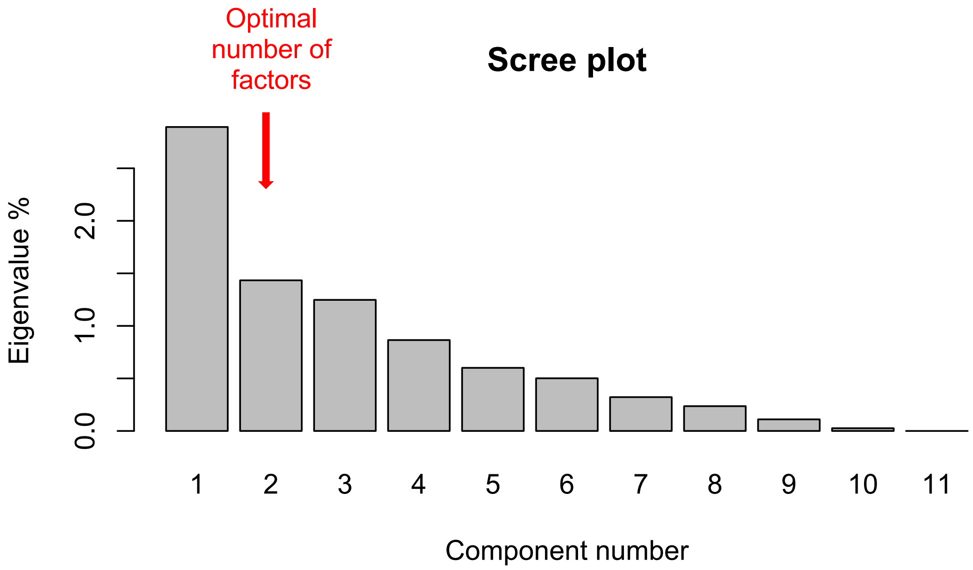

C/ Reaching a reliable and fitted factor solution

We used an iterative process of removal of items according to the following rule: at each iteration, we identified the items with no loading >.30 on any factor and we removed the item with the lowest loadings until all the items showed one loading >.30 on one factor.

In the initial result of the EFA, two items had no loading >.30 on any factor (see table below). After removing the item with the lowest loading on F1 (i.e., $\text{Delta}_{(FP\text{-}GP)}$ Walking occurrences), we reached a factor solution in which all items had one loading >.30 on one factor (see final solution in Table 2 in the manuscript).

Observed variables	Factor loadings	
	F1	F2
SAS score	**0.85**	0.28
DAS-Executive score	**0.63**	0.14
$\text{Mean}_{(FP+GP)}$ Activity time ratio	**-0.53**	0.25
$\text{Mean}_{(FP+GP)}$ Walking acceleration	**-0.35**	0.02
$\text{Mean}_{(FP+GP)}$ Walking duration	**0.42**	0.07
$\text{Mean}_{(FP+GP)}$ Walking occurrences	0.27	-0.01
$\text{Delta}_{(FP\text{-}GP)}$ Walking occurrences	0.24	-0.01
DAS-Initiation score	0.20	**0.68**
$\text{Delta}_{(FP\text{-}GP)}$ Walking duration	-0.11	**0.62**
DAS-Emotional score	0.12	**0.62**
$\text{Delta}_{(FP\text{-}GP)}$ Walking acceleration	0.08	**-0.49**

Using this method of item removal allowed to reach a well-fitted and reliable final two-factor model. The SRMR and the RMSEA correspond to the average difference between the sample variance/covariance matrix and the variance/covariance matrix estimated by the model. A satisfactory fit is indicated by an SRMR value ≤ 0.08 and an RMSEA value ≤ 0.05. For our final solution, we found that SRMR = 0.08 and RMSEA = 0.03. The reliability of measures by factors can be assessed using Cronbach's alpha (α) with a minimum threshold of .60. With the final factor solution, we found that α= .62 for F1 dimension and α= .60 for F2 dimension.

D/ Theoretical arguments justifying the removal of two items

Two calculated deltas were excluded from the final factor solution: the delta of activity time ratio and the delta of walking occurrences. From a theoretical point of view, these two deltas present specificities which could explain that they are not related to the deficit of self-initiation. These two deltas compare metrics which are not exactly equivalent (and therefore comparable) in the free and guided phase, in terms of quantification of goal-directed behaviors.

Regarding the activity time ratio, the measure in the free phase only depends on the capacity to initiate and maintain goal-directed behaviors. In the guided phase, the performative ability to complete the questionnaire more or less quickly is another parameter which can impact the measure of the activity time ratio (aside from the strict capacity to initiate and maintain goal-directed behaviors).

Regarding walking occurrences, in the free phase, they are related to higher wandering in the room and supposedly to lower ability to initiate and maintain static goal-directed behaviors (e.g., reading, playing games etc…). In the guided phase, walking initiation can be at the expense of focusing on goal-directed behaviors but can also serve the purpose of completing the questionnaire (which require exploration of the room). We also observed that bvFTD patients walked more frequently than controls only in the free phase and walking occurrences were more closely related to apathy (measured by questionnaire) in the free phase. This further confirmed that walking occurrences were probably better quantifiers of the reduction of goal-directed behaviors in the free phase than in the guided phase.
