# Supplementary material for: Functional connectivity correlates of reduced goal-directed behaviors in behavioural variant frontotemporal dementia
Source: Brain Struct Funct. 2022 Jun 25;227(9):2971–89. doi: 10.1007/s00429-022-02519-5 (PMC9653340; doi:10.1007/s00429-022-02519-5)

Supplementary file 4

Effect sizes and group specificities for functional connectivity analyses (fALFF and seed-based) related to apathy dimensions

First, for each reported result (fALFF and seed-based), the effect size (i.e., Fisher-transformed correlation coefficient) with confidence interval is displayed for all the identified significant clusters (detailed in Table 3 for fALFF results and in Table 4 for seed-based results). *NB*: clusters in brain stem and in cerebellum are not visible in the figures which show projections onto cortical surface.

Second, for each reported result (fALFF and seed-based), the specificities of the relationship between F1/F2 and functional activity/connectivity within each group (bvFTD and controls) were explored. We could not evidence any significant cluster by testing the difference of effect of F1/F2 between bvFTD and controls, nor by testing the effect of F1/F2 separately within each group (probably due to lack of statistical power). Therefore, to account for the specific characteristics of each group, we show for each reported analysis: 1/ a scatterplot showing the correlation, within the bvFTD group and within the control group, between F1/F2 and the measure of functional activity/connectivity in one significant cluster; 2/ the uncorrected raw T-maps for the test of the effect of F1/F2 separately within each group.

Finally, for each reported result, we tested again the effect of F1/F2 after taking account of the clinical status (bvFTD vs control) in the model (along with age and sex). Results with identified significant clusters are provided in section C/ of this document.

A/ fALFF and apathy

- Effect of F1 on whole-brain low-frequency signal power (fALFF)

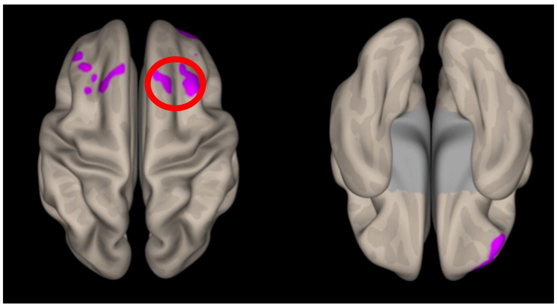

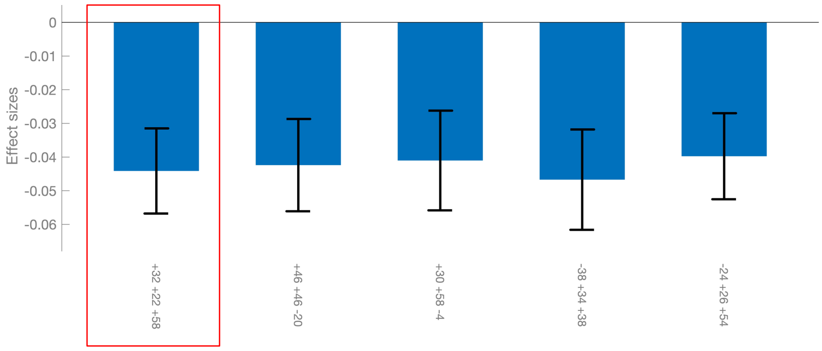

- Correlation between F1 and fALFF in the cluster circled in red in bvFTD patients (in blue) and in controls (in yellow)

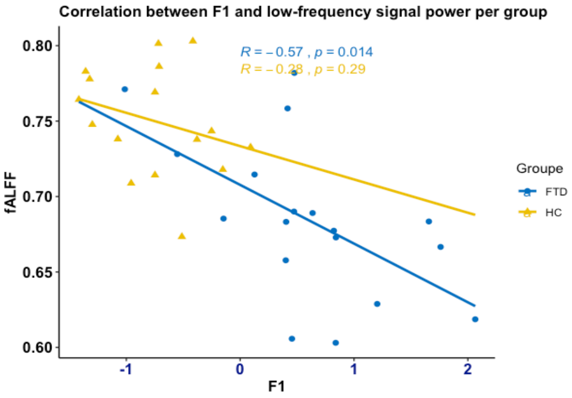

- Uncorrected T-maps for the test of the effect of F1 on whole-brain fALFF within bvFTD group (on the left) and within control group (on the right)

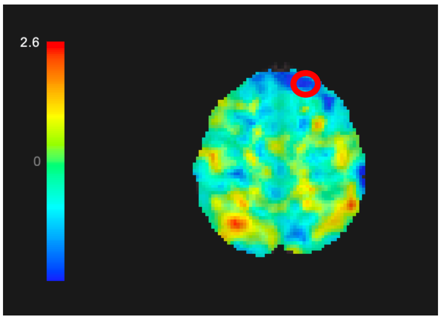

x,y,z=(32,22,58)mm
T=-2.98 ; p< .01

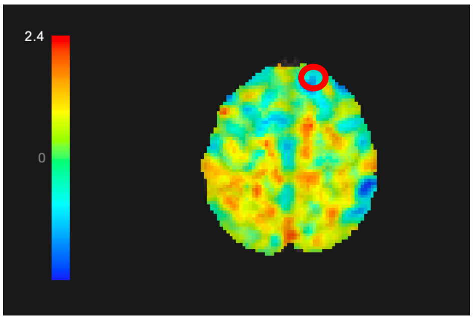

x,y,z=(32,22,58)mm
T=-0.80 ; p= .43

B/ Seed-based connectivity and apathy

- Effect of F1 on seed-based connectivity of ACC (SN hub)

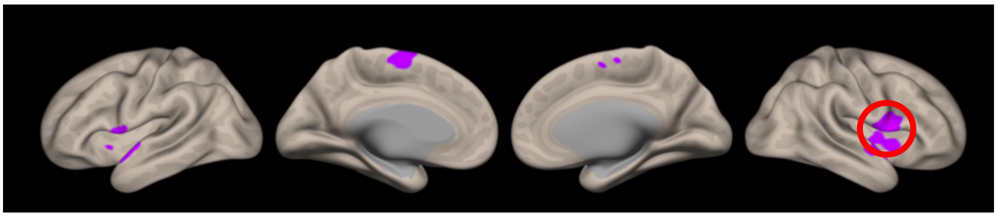

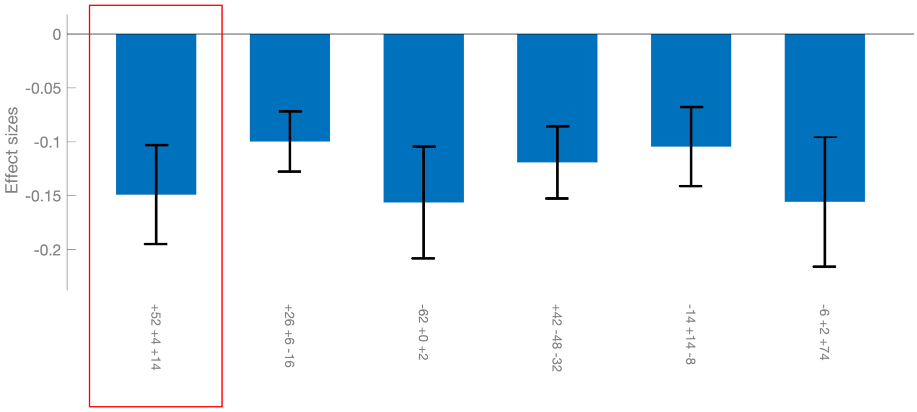

- Correlation between F1 and connectivity with ACC in the cluster circled in red in bvFTD patients (in blue) and in controls (in yellow)

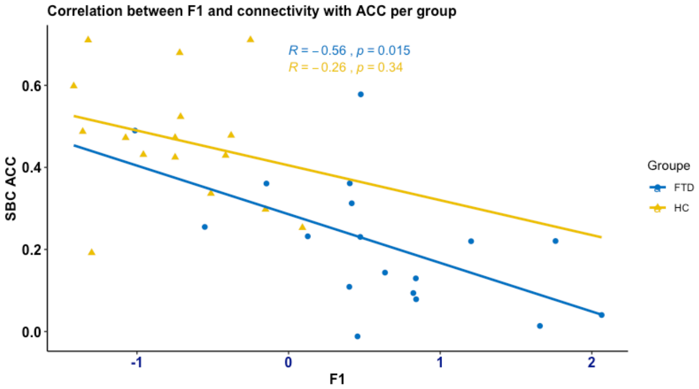

- Uncorrected T-maps for the test of the effect of F1 on seed-based connectivity of ACC within bvFTD group (on the left) and within control group (on the right)

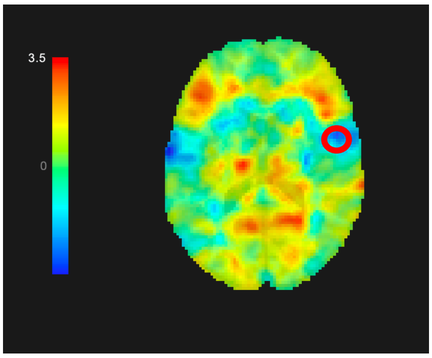

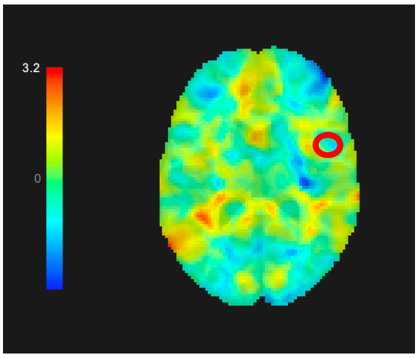

x,y,z=(52,4,14)mm
T=-3.70 ; p< .001

x,y,z=(52,4,14)mm
T=-1.74 ; p= .09

- Effect of F1 on seed-based connectivity of left AI (SN hub)

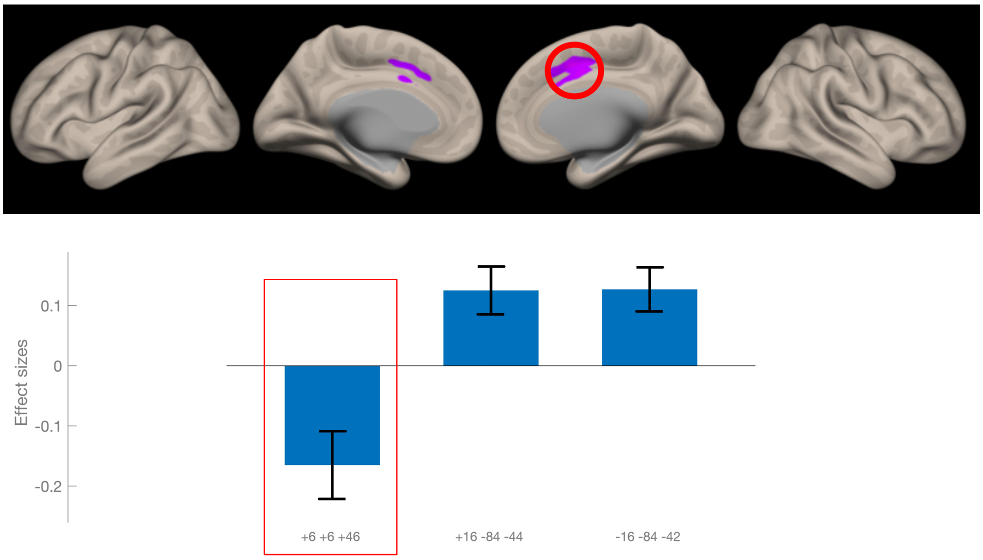

- Correlation between F1 and connectivity with left AI in the cluster circled in red in bvFTD patients (in blue) and in controls (in yellow)

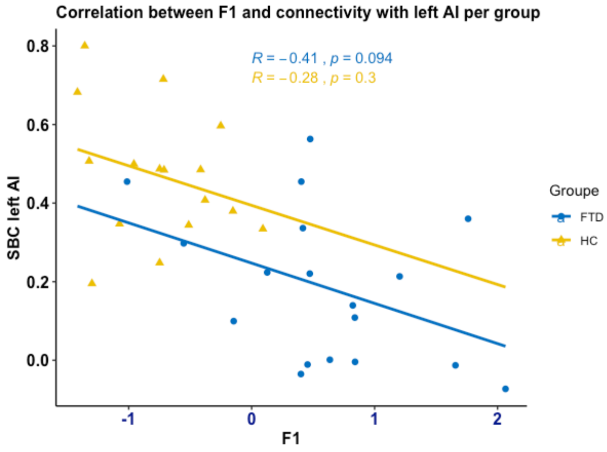

- Uncorrected T-maps for the test of the effect of F1 on seed-based connectivity of left AI within bvFTD group (on the left) and within control group (on the right)

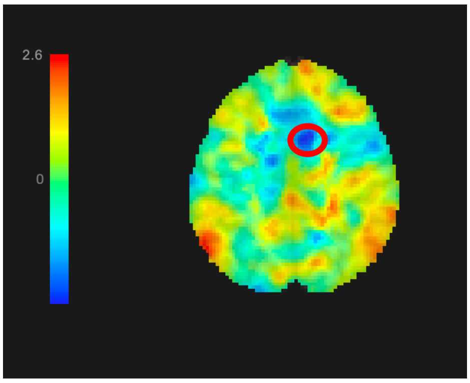

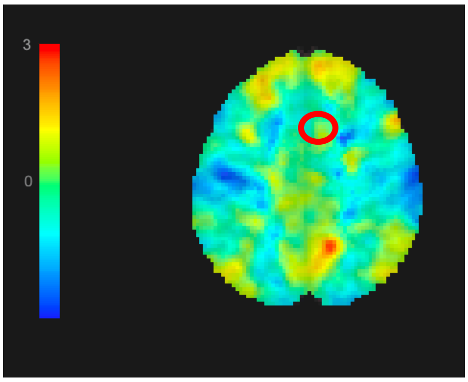

x,y,z=(6,6,46)mm
T=-2.61 ; p= .01

x,y,z=(6,6,46)mm
T=0.15 ; p= .88

- Effect of F1 on seed-based connectivity of right AI (SN hub)

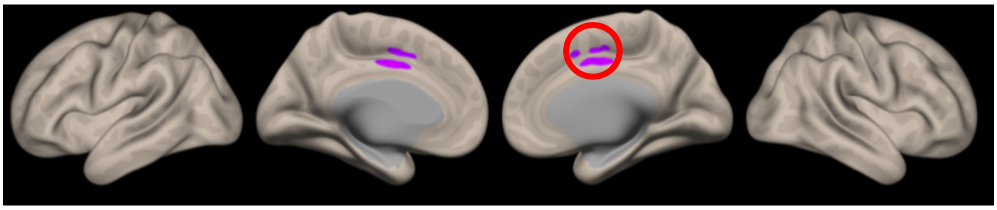

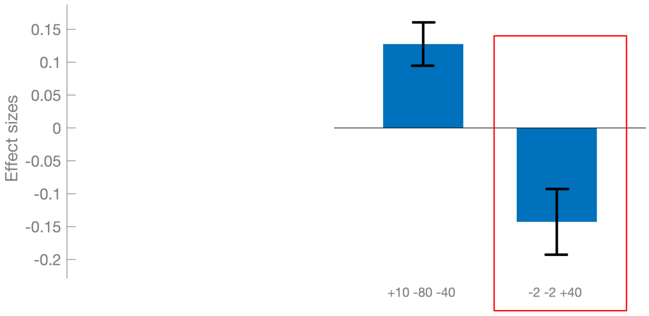

- Correlation between F1 and connectivity with right AI in the cluster circled in red in bvFTD patients (in blue) and in controls (in yellow)

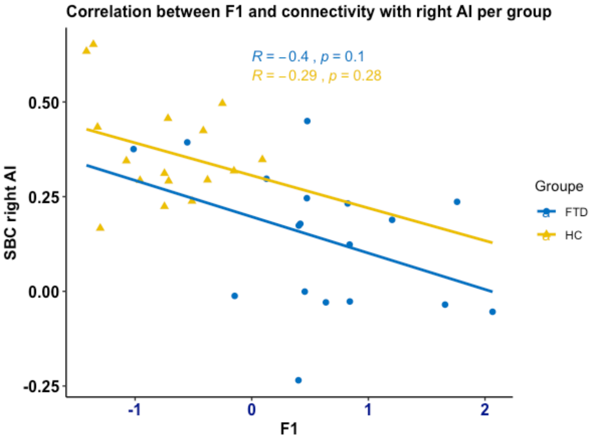

- Uncorrected T-maps for the test of the effect of F1 on seed-based connectivity of right AI within bvFTD group (on the left) and within control group (on the right)

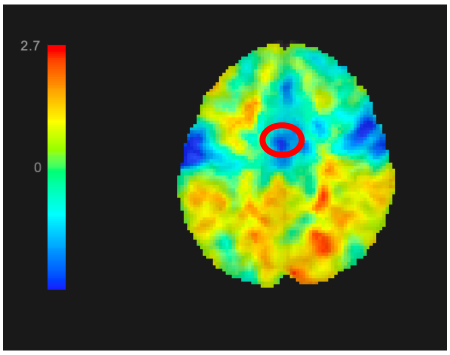

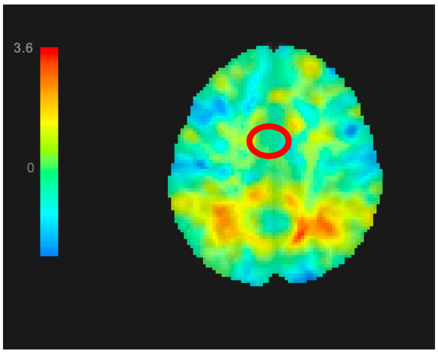

x,y,z=(6,6,46)mm
T=-2.76 ; p< .01

x,y,z=(6,6,46)mm
T=-0.60 ; p= .55

- Effect of F2 on seed-based connectivity of left LPC (DMN hub)

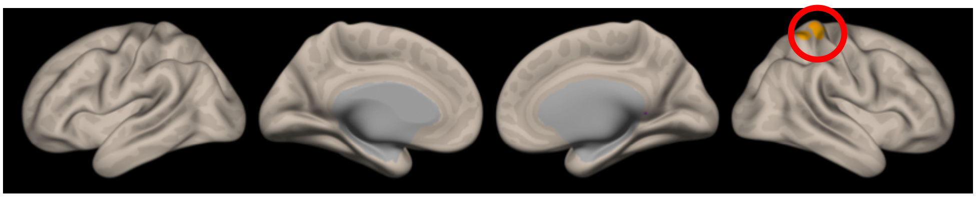

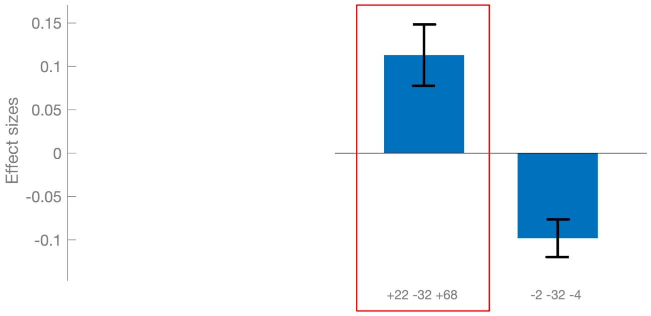

- Correlation between F2 and connectivity with left LPC in the cluster circled in red in bvFTD patients (in blue) and in controls (in yellow)

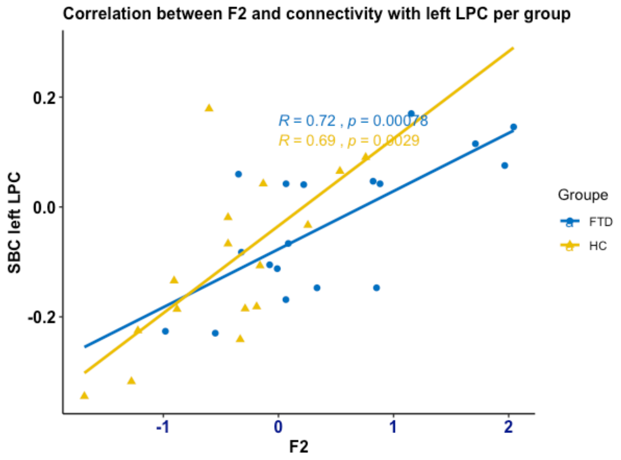

- Uncorrected T-maps for the test of the effect of F2 on seed-based connectivity of left LPC within bvFTD group (on the left) and within control group (on the right)

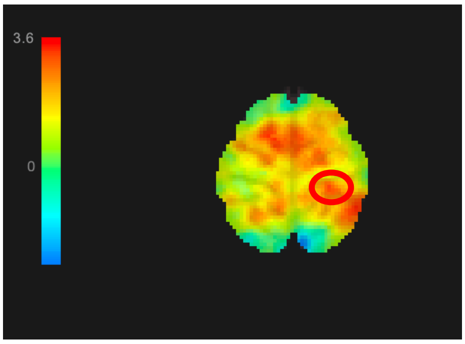

x,y,z=(22,-32,68)mm
T=3.43 ; p< .01

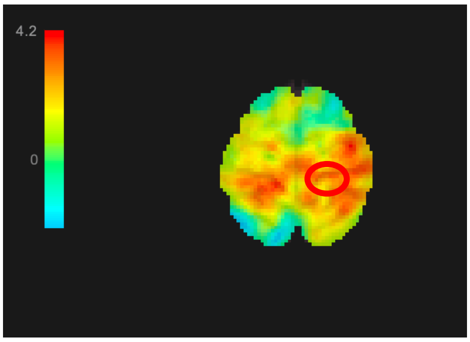

x,y,z=(22,-32,68)mm
T=2.91 ; p< .01

- Effect of F2 on seed-based connectivity of right LPC (DMN hub)

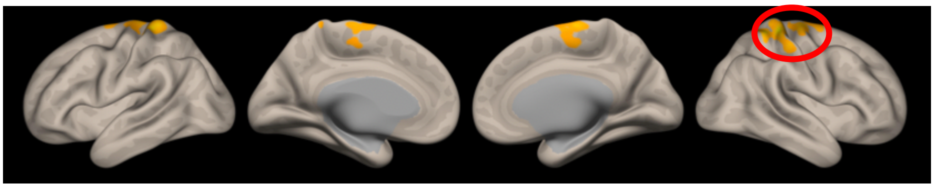

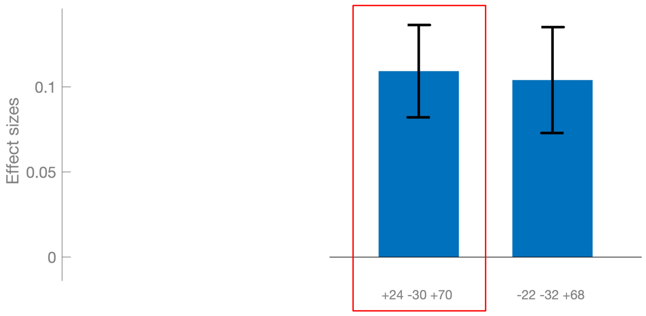

- Correlation between F2 and connectivity with right LPC in the cluster circled in red in bvFTD patients (in blue) and in controls (in yellow)

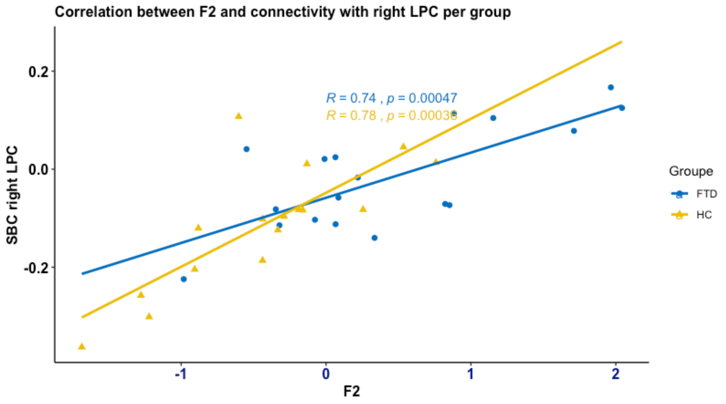

- Uncorrected T-maps for the test of the effect of F2 on seed-based connectivity of right LPC within bvFTD group (on the left) and within control group (on the right)

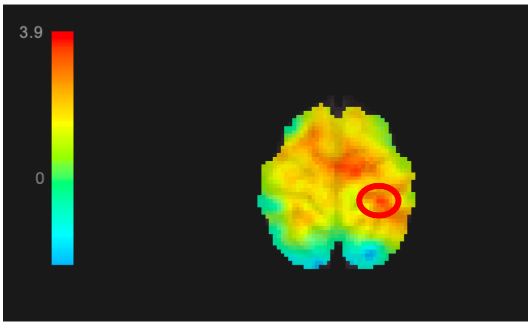

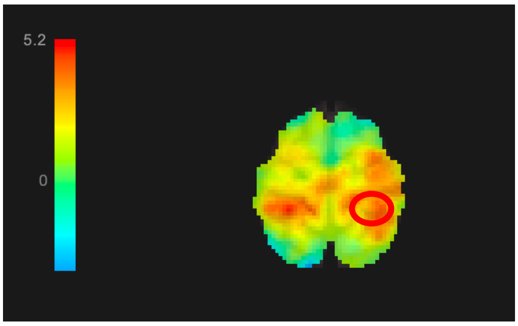

x,y,z=(24,-30,70)mm
T=3.91 ; p< .001

x,y,z=(24,-30,70)mm
T=4.08 ; p< .001

C/ Effect of apathy dimensions on connectivity controlling for clinical status

We found significant clusters for:

- The effect of F2 on the seed-based connectivity of left LPC

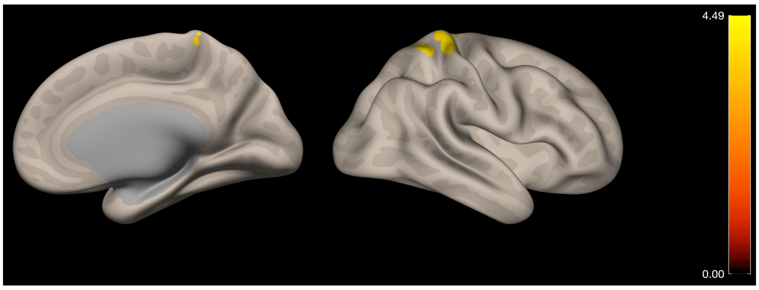

- The effect of F2 on the seed-based connectivity of right LPC

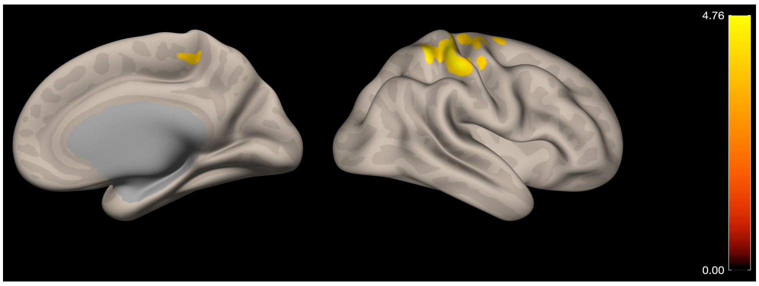

Supplement: Supplementary file 4 — Supplementary file4 (PDF 18066 kb) [file 429_2022_2519_MOESM4_ESM.pdf]
